# Consumption of coffee and tea with all-cause and cause-specific mortality: a prospective cohort study

**DOI:** 10.1186/s12916-022-02636-2

**Published:** 2022-11-18

**Authors:** Yanchun Chen, Yuan Zhang, Mengnan Zhang, Hongxi Yang, Yaogang Wang

**Affiliations:** 1 School of Public Health, Tianjin Medical University, Qixiangtai Road 22, Heping District, Tianjin, 300070 China; 2 School of Basic Medical Sciences, Tianjin Medical University, Qixiangtai Road 22, Heping District, Tianjin, 300070 China

**Keywords:** Coffee consumption, Tea consumption, All-cause mortality, Cause-specific mortality, Prospective study

## Abstract

**Background:**

Previous studies suggested that moderate coffee and tea consumption are associated with lower risk of mortality. However, the association between the combination of coffee and tea consumption with the risk of mortality remains unclear. This study aimed to evaluate the separate and combined associations of coffee and tea consumption with all-cause and cause-specific mortality.

**Methods:**

This prospective cohort study included 498,158 participants (37–73 years) from the UK Biobank between 2006 and 2010. Coffee and tea consumption were assessed at baseline using a self-reported questionnaire. All-cause and cause-specific mortalities, including cardiovascular disease (CVD), respiratory disease, and digestive disease mortality, were obtained from the national death registries. Cox regression analyses were conducted to estimate hazard ratios (HRs) and 95% confidence intervals (CIs).

**Results:**

After a median follow-up of 12.1 years, 34,699 deaths were identified. The associations of coffee and tea consumption with all-cause and cause-specific mortality attributable to CVD, respiratory disease, and digestive disease were nonlinear (all *P* nonlinear < 0.001). The association between separate coffee consumption and the risk of all-cause mortality was J-shaped, whereas that of separate tea consumption was reverse J-shaped. Drinking one cup of coffee or three cups of tea per day seemed to link with the lowest risk of mortality. In joint analyses, compared to neither coffee nor tea consumption, the combination of < 1–2 cups/day of coffee and 2–4 cups/day of tea had lower mortality risks for all-cause (HR, 0.78; 95% CI: 0.73–0.85), CVD (HR, 0.76; 95% CI: 0.64–0.91), and respiratory disease (HR, 0.69; 95% CI: 0.57–0.83) mortality. Nevertheless, the lowest HR (95% CI) of drinking both < 1–2 cup/day of coffee and ≥ 5 cups/day of tea for digestive disease mortality was 0.42 (0.34–0.53).

**Conclusions:**

In this large prospective study, separate and combined coffee and tea consumption were inversely associated with all-cause and cause-specific mortality.

**Supplementary Information:**

The online version contains supplementary material available at 10.1186/s12916-022-02636-2.

## Background

Globally, coffee and tea are among the most widely consumed beverages and become part of people’s dietary patterns [1]. Epidemiologic studies have found that separate coffee and tea consumption were associated with non-communicable diseases, like cardiovascular disease (CVD), type 2 diabetes, and esophageal cancer [2–4]. Accumulating evidence suggests that separate coffee and tea consumption are associated with mortality [5–10]; however, the shape of the association is controversial, since studies have found that there are different types of U-shaped, J-shaped, linear, or null associations between separate coffee and tea consumption and mortality [6–8]. Montagnana et al. reported a U-shaped association between coffee and mortality, with the highest risk at intermediate consumption (2 to 4 cups/day) [9], while another study revealed that coffee consumption was inversely associated with mortality even among those who drank 8 or more cups/day [10]. Given coffee and tea drinking are often considered unhealthy lifestyles because of the caffeine they contain [11], identifying the effect of coffee and tea on mortality requires further study.

Of note, emerging evidence has confirmed the interaction between coffee and tea with lower risks of serious diseases such as stroke and dementia [12, 13]. Up to now, only one cohort study from the Japanese population has examined the combination of coffee and tea consumption with the risk of mortality [14], and it was found that the combined effect of green tea and coffee on mortality appeared to be additive in patients with type 2 diabetes. However, this study is limited by the small sample size, single evaluation of covariates (lifestyle, diet), and insufficient adjustment for important confounding factors. Additionally, it remains unclear whether such findings apply to other populations with different genetic and environmental backgrounds.

Therefore, this study aimed to examine the separate and combined associations of coffee and tea consumption with total and cause-specific mortality (including cardiovascular disease [CVD], respiratory disease, and digestive disease) using data from a population-based longitudinal cohort of UK Biobank. Furthermore, we aimed to conduct stratification analyses according to important baseline factors, including various lifestyles and the presence or absence of chronic diseases, to examine whether the association between mortality and the joint exposures of coffee and tea varied by these factors.

## Methods

### Study design and population

The UK Biobank is a large-scale prospective study that recruited 502,507 participants aged 37–73 years from the general population between 2006 and 2010 [15]. Participants attended 1 of 22 dedicated assessment centers nationally across England, Wales, and Scotland where they provided information on health-related aspects through touch-screen questionnaires and physical measurements [16]. The details of the study design and methods have been described in previous studies [17]. In the present study, we excluded participants who were lost to follow-up (*n* = 1346) or with missing information on coffee or tea consumption (*n* = 3003) at baseline, leaving 498,158 participants for the primary analysis (Additional file 1: Fig. S1).

### Assessment of coffee and tea consumption

The touchscreen questionnaire included part of a dietary assessment of a series of common food and beverage items. Participants were asked about their average intake of coffee in the last year “How many cups of coffee do you drink each day (including decaffeinated coffee)?” and “How many cups of tea do you drink each day (including black and green tea)?” Participants either selected the number of cups, “Less than 1,” “Do not know,” or “Prefer not to answer.” If coffee and tea consumption exceeded 10 and 20 cups/day, respectively, then participants were asked to confirm their answers.

### Assessment of covariates

To guide covariates selection, we constructed a directed acyclic graph (DAG) based on sociodemographic characteristics and a prior knowledge of potential confounding factors associated with all-cause mortality [10, 18, 19]. Additional file 1: Fig. S2 shows the DAG depicted causal relationships between measured variables in the current analysis. The program of DAGitty was used to identify the minimally sufficient adjustment set [20]. We used the baseline touch-screen questionnaire to collect sociodemographic, behavioral, and other factors. Sociodemographic factors were documented including sex, age, ethnicity (White, Asian or Asian British, Black or Black British, and others), and education levels (college or university degree, upper secondary, lower secondary, vocational, and others). Behavioral factors included smoking status (never, previous, and current), alcohol intake frequency (never, special occasions only, one to three times a month, once or twice a week, three or four times a week, daily or almost daily), physical activity (low, middle, and high, measured using the International Physical Activity Questionnaire [IPAQ]), and dietary pattern (healthy and unhealthy, healthy diet was based on consumption of at least 4 of 7 dietary components: fruits: ≥ 3 servings/day, vegetables: ≥ 3 servings/day, fish: ≥ 2 servings/week, processed meats: ≤ 1 serving/week, unprocessed red meats: ≤ 1.5 servings/week, whole grains: ≥ 3 servings/day, refined grains: ≤ 1.5 servings/day) (Additional file 1: Table S1) [21, 22]. Body mass index (BMI) (< 25, 25 to < 30, ≥ 30 kg/m^2^) was derived from physical measurement and calculated by dividing weight (kg) over height (m) squared. General health status was categorized as excellent, good, fair, and poor. Information on chronic diseases (e.g., hypertension, diabetes, and depression) was collected from touchscreen questionnaires, medical examinations, and hospital inpatient records.

### Ascertainment of outcomes

Mortality information was obtained from death certificates, which were provided by the NHS Information Centre (England and Wales) and the NHS Central Register Scotland (Scotland) for the date of death. International Classification of Diseases (ICD-10) codes were used to classify deaths from CVD (ICD 10 codes I00-I79), respiratory disease (ICD 10 codes J09-J18 and J40-J47), digestive disease (ICD 10 codes K20-K93), and other causes.

### Statistical analyses

We summarized baseline characteristics according to coffee and tea consumption categories as percentages for categorical variables, while means with standard deviations (SDs) for normal continuous variables and median and interquartile range (IQR) for non-normal variables. Normality test was applied by Shapiro-Wilk normality test. Multiple imputations using the chained equations (MICE) method were performed to handle missing covariates. Five imputed datasets were constructed and Rubin’s rule was used to combine the results [23].

To assess the dose-response associations of separate coffee and tea consumption with all-cause mortality and cause-specific mortality, we used restricted cubic splines models with 4 knots at the 25th, 50th, 75th, and 95th centiles. Tests for linearity or nonlinearity used the Wald test to calculate *P*-values, which was performed to test the null hypothesis that the coefficient of the second spline is equal to 0 [24]. In our analysis, the null hypothesis was rejected (*P* < 0.05) and concluded that there was a nonlinear relationship between separate coffee and tea consumption with all-cause mortality and cause-specific mortality. In the spline models, we adjusted for potential confounders including sex, age, ethnicity, education levels, BMI, smoking status, alcohol intake frequency, physical activity, dietary pattern, general health status, hypertension, diabetes, and depression. Coffee and tea consumption were mutually adjusted. Then, we divided coffee and tea consumption into four groups using prior validated thresholds based on the restricted cubic spline of association between separate consumption of coffee and tea with mortality. Participants who drank ≥ 5 cups/day of coffee or tea were defined as excess consumption based on previous studies [25]. Finally, we defined the categories as follows: coffee: none, < 1–2, 3–4, and ≥ 5 cups/day; tea: none, < 1–1, 2–4, and ≥ 5 cups/day. Cox proportional hazards models were used to estimate the hazard ratios (HRs) and 95% confidence intervals (CIs) of separate coffee and tea consumption groups with all-cause mortality and cause-specific mortality. Proportional hazard assumptions were verified using the Schoenfeld residuals method, and no significant deviations were observed. Follow-up time was calculated from the date of questionnaire completion in which the baseline coffee and tea consumption were available, lost to follow-up, death, or end of follow-up (23 March 2021), whichever came first. The multi-adjusted models were adjusted with the same covariates as the restricted cubic spline. Furthermore, to quantify the magnitude of combined consumption of coffee and tea with mortality, participants were categorized into 16 groups according to coffee and tea consumption categories, with participants who had neither coffee nor tea consumption comprising the reference group. Coffee consumption of < 1–2 cups/day and tea consumption of 2–4 cups/day were combined into one category because these participants had the highest proportion and the lowest mortality rate.

In addition, we performed subgroup analyses to assess potential modification effects and determine whether there was any population heterogeneity according to age, sex, BMI, physical activity, smoking status, alcohol intake frequency, dietary pattern, hypertension, diabetes, and depression. The interactions between baseline characteristics and combined coffee and tea consumption (combined < 1–2 cups/day of coffee and 2–4 cups/day of tea vs. neither coffee nor tea consumption) were examined using the likelihood ratio test (LRT).

### Sensitivity analysis

Additional analyses were further conducted. First, we repeated the main analyses by excluding mortality cases that occurred in the first 3 years of follow-up. Second, we conducted the main analyses using available data before multiple imputations for missing covariates. Third, because severe diseases could confound results, we defined a population that excluded participants with prevalent CVD and cancer at baseline. Fourth, smoking may have potential effect modification because there may be unmeasured confounders between previous and current smokers. Therefore, we repeated the main analyses adjusted for pack-years categories of cigarette smoking (nonsmokers: having smoked zero pack-years; light smokers: fewer than 20 pack-years; and heavy smokers: 20 or more pack-years). Pack-years of cigarette smoking were calculated as the number of cigarettes smoked per day divided by 20 and then multiplied by the number of years of smoking [26]. Fifth, the information on both coffee and tea consumption and potential confounders were collected at the baseline; it is very difficult to assess the role of depression in these associations. We performed main analyses without adjusting for baseline depression. Furthermore, we also investigated coffee and tea consumption interaction on mortality in the above sensitivity analyses. All analyses were performed using R (version 3.6.3, R Foundation for Statistical Computing) and STATA 15 statistical software (StataCorp). The two-sided *P* < 0.05 was considered statistically significant.

## Results

### Characteristics of the study population

In total, 498,158 participants (median (IQR) age: 58 [50-63]; 54% female) were included in this analysis. The proportion of < 1–2 cups/day of coffee and 2–4 cups/day of tea were 46.1% (*n* = 229,498) and 43.7% (*n* = 217,454), respectively. Additional file 1: Fig. S3 shows the distribution for the combination of coffee and tea consumption. Drinking both < 1–2 cups/day of coffee and 2–4 cups/day of tea was more prevalent accounting for the largest proportion with 23.0% (*n* = 114,629). Table 1 presents the baseline characteristics of participants according to coffee and tea consumption. Compared with non-coffee consumers, those who consumed < 1–2 cups/day of coffee were more likely to be older, male, white, non-drinker, more likely to have a university education level, high physical activity, healthy diet, and more likely to report “excellent” health; but they were less likely to be obese, non-smoker, and less likely to have diabetes, hypertension, and depression. Likewise, as compared to non-tea consumers, those who consumed 2–4 cups/day of tea were more likely to be older, male, white, non-drinker, more likely to have high physical activity, healthy diet, hypertension, and more likely to report “excellent” health, but they were less likely to be obese, non-smoker, and less likely to have a university education level, diabetes, and depression. Over 15 years of follow-up (median [IQR] length of follow-up, 12.1 [11.4–12.8] years; total person-years, 5,900,033), we documented 34,699 (7.0%) deaths. Among them, 6663 (1.3%) participants died from CVD, 6018 (1.2%) participants died from respiratory disease, and 2864 (0.6%) participants died from digestive disease.

**Table 1:** Baseline characteristics according to coffee and tea consumption

Characteristic	Coffee consumption (cups/day)				Tea consumption (cups/day)			
	None	< 1–2	3–4	≥ 5	None	< 1–1	2–4	≥ 5
Total	110,646 (22.2)	229,498 (46.1)	102,694 (20.6)	55,320 (11.1)	73,223 (14.7)	57,438 (11.5)	217,454 (43.7)	150,043 (30.1)
Age, median (IQR), year	56 (49–62)	59 (51–64)	58 (51–63)	57 (49–62)	56 (48–62)	56 (48–63)	58 (51–64)	58 (51–63)
Female	64,412 (58.2)	127,729 (55.7)	52,750 (51.4)	26,246 (47.4)	41,350 (56.5)	29,958 (52.2)	118,940 (54.7)	80,889 (53.9)
Education level								
College/university degree	30,021 (27.1)	79,979 (34.8)	36,493 (35.5)	16,436 (29.7)	23,198 (40.4)	73,844 (34.0)	43,535 (29.0)	23,198 (40.4)
Upper secondary	11,763 (10.6)	26,613 (11.6)	11,711 (11.4)	5997 (10.8)	7211 (12.6)	24,543 (11.3)	15,816 (10.5)	7211 (12.6)
Lower secondary	31,333 (28.3)	59,743 (26.0)	26,955 (26.2)	15,758 (28.5)	14,320 (24.9)	57,810 (26.6)	40,350 (26.9)	14,320 (24.9)
Vocational	7923 (7.2)	14,327 (6.2)	6585 (6.4)	4248 (7.7)	3186 (5.5)	13,755 (6.3)	11,222 (7.5)	3186 (5.5)
Others	29,606 (26.8)	48,836 (21.3)	20,950 (20.4)	12,881 (23.3)	9523 (16.6)	47,502 (21.8)	39,120 (26.1)	9523 (16.6)
Ethnicity								
White	99,782 (90.2)	217,514 (94.8)	100,110 (97.5)	54,241 (98.0)	52,829 (92.0)	203,056 (93.4)	145,716 (97.1)	52,829 (92.0)
Asian/Asian British	5203 (4.7)	4910 (2.1)	807 (0.8)	281 (0.5)	1649 (2.9)	6928 (3.2)	1753 (1.2)	1649 (2.9)
Black/Black British	3417 (3.1)	3498 (1.5)	730 (0.7)	261 (0.5)	1642 (2.9)	3991 (1.8)	1005 (0.7)	1642 (2.9)
Others	2244 (2.0)	3576 (1.6)	1047 (1.0)	537 (1.0)	1318 (2.3)	3479 (1.6)	1569 (1.0)	1318 (2.3)
BMI (kg/m^2^)								
< 25	36,251 (32.8)	80,076 (34.9)	30,886 (30.1)	14,455 (26.1)	18,952 (33.0)	73,452 (33.8)	48,043 (32.0)	18,952 (33.0)
25 to < 30	45,545 (41.2)	97,643 (42.5)	45,602 (44.4)	24,213 (43.8)	24,148 (42.0)	93,778 (43.1)	65,325 (43.5)	24,148 (42.0)
≥ 30	28,850 (26.1)	51,779 (22.6)	26,206 (25.5)	16,652 (30.1)	14,338 (25.0)	50,224 (23.1)	36,675 (24.4)	14,338 (25.0)
Physical activity								
Low	22,579 (20.4)	41,187 (17.9)	19,398 (18.9)	11,907 (21.5)	11,486 (20.0)	40,070 (18.4)	28,267 (18.8)	11,486 (20.0)
Middle	43,656 (39.5)	95,257 (41.5)	42,526 (41.4)	21,547 (38.9)	23,770 (41.4)	90,245 (41.5)	59,957 (40.0)	23,770 (41.4)
High	44,411 (40.1)	93,054 (40.5)	40,770 (39.7)	21,866 (39.5)	22,182 (38.6)	87,139 (40.1)	61,819 (41.2)	22,182 (38.6)
Smoking status								
Never	65,038 (58.8)	130,528 (56.9)	53,733 (52.3)	23,467 (42.4)	31,320 (54.5)	123,264 (56.7)	80,012 (53.3)	31,320 (54.5)
Previous	34,794 (31.4)	80,371 (35.0)	37,635 (36.6)	19,961 (36.1)	19,840 (34.5)	76,086 (35.0)	51,831 (34.5)	19,840 (34.5)
Current	10,814 (9.8)	18,599 (8.1)	11,326 (11.0)	11,892 (21.5)	6278 (10.9)	18,104 (8.3)	18,200 (12.1)	6278 (10.9)
Alcohol intake frequency								
Never	16,033 (14.5)	49,119 (21.4)	24,778 (24.1)	11,460 (20.7)	15,204 (20.8)	14,410 (25.1)	46,723 (21.5)	25,053 (16.7)
Special occasions only	19,469 (17.6)	56,404 (24.6)	26,768 (26.1)	12,304 (22.2)	14,603 (19.9)	13,544 (23.6)	53,378 (24.5)	33,420 (22.3)
One to three times a month	27,509 (24.9)	60,925 (26.5)	26,177 (25.5)	14,066 (25.4)	17,602 (24.0)	13,512 (23.5)	56,771 (26.1)	40,792 (27.2)
Once or twice a week	13,926 (12.6)	24,886 (10.8)	10,223 (10.0)	6531 (11.8)	8482 (11.6)	5938 (10.3)	22,641 (10.4)	18,505 (12.3)
Three or four times a week	17,582 (15.9)	24,070 (10.5)	9239 (9.0)	6634 (12.0)	9647 (13.2)	5940 (10.3)	22,339 (10.3)	19,599 (13.1)
Daily or almost daily	16,127 (14.6)	14,094 (6.1)	5509 (5.4)	4325 (7.8)	7685 (10.5)	4094 (7.1)	15,602 (7.2)	12,674 (8.4)
Self-reported health								
Excellent	15,864 (14.3)	39,346 (17.1)	18,172 (17.7)	8283 (15.0)	11,834 (16.2)	10,433 (18.2)	36,796 (16.9)	22,602 (15.1)
Good	61,382 (55.5)	135,704 (59.1)	60,741 (59.1)	30,764 (55.6)	40,536 (55.4)	33,089 (57.6)	129,023 (59.3)	85,943 (57.3)
Fair	26,455 (23.9)	45,511 (19.8)	20,069 (19.5)	13,108 (23.7)	16,616 (22.7)	11,457 (19.9)	43,352 (19.9)	33,718 (22.5)
Poor	6945 (6.3)	8937 (3.9)	3712 (3.6)	3165 (5.7)	4237 (5.8)	2459 (4.3)	8283 (3.8)	7780 (5.2)
Diet pattern								
Unhealthy	62,139 (56.2)	127,531 (55.6)	58,913 (57.4)	33,628 (60.8)	42,795 (58.4)	33,271 (57.9)	120,774 (55.5)	85,371 (56.9)
Healthy	48,507 (43.8)	101,967 (44.4)	43,781 (42.6)	21,692 (39.2)	30,428 (41.6)	24,167 (42.1)	96,680 (44.5)	64,672 (43.1)
Diabetes								
No	104,157 (94.1)	218,198 (95.1)	97,350 (94.8)	52,086 (94.2)	68,596 (93.7)	54,341 (94.6)	206,292 (94.9)	142,562 (95.0)
Yes	6489 (5.9)	11,300 (4.9)	5344 (5.2)	3234 (5.8)	4627 (6.3)	3097 (5.4)	11,162 (5.1)	7481 (5.0)
Hypertension								
No	79,133 (71.5)	166,543 (72.6)	75,328 (73.4)	40,832 (73.8)	53,371 (72.9)	42,392 (73.8)	157,500 (72.4)	108,573 (72.4)
Yes	31,513 (28.5)	62,955 (27.4)	27,366 (26.6)	14,488 (26.2)	19,852 (27.1)	15,046 (26.2)	59,954 (27.6)	41,470 (27.6)
Depression								
No	101,860 (92.1)	216,965 (94.5)	97,530 (95.0)	51,269 (92.7)	68,280 (93.2)	53,979 (94.0)	205,218 (94.4)	140,147 (93.4)
Yes	8786 (7.9)	12,533 (5.5)	5164 (5.0)	4051 (7.3)	4943 (6.8)	3459 (6.0)	12,236 (5.6)	9896 (6.6)

*Abbreviations*: *BMI* body mass index (calculated as weight in kilograms divided by height in meters squared); *IQR* interquartile range (75th quartile minus 25th quartile)

### Nonlinear association between separate coffee and tea consumption with mortality

**Fig. 1:**
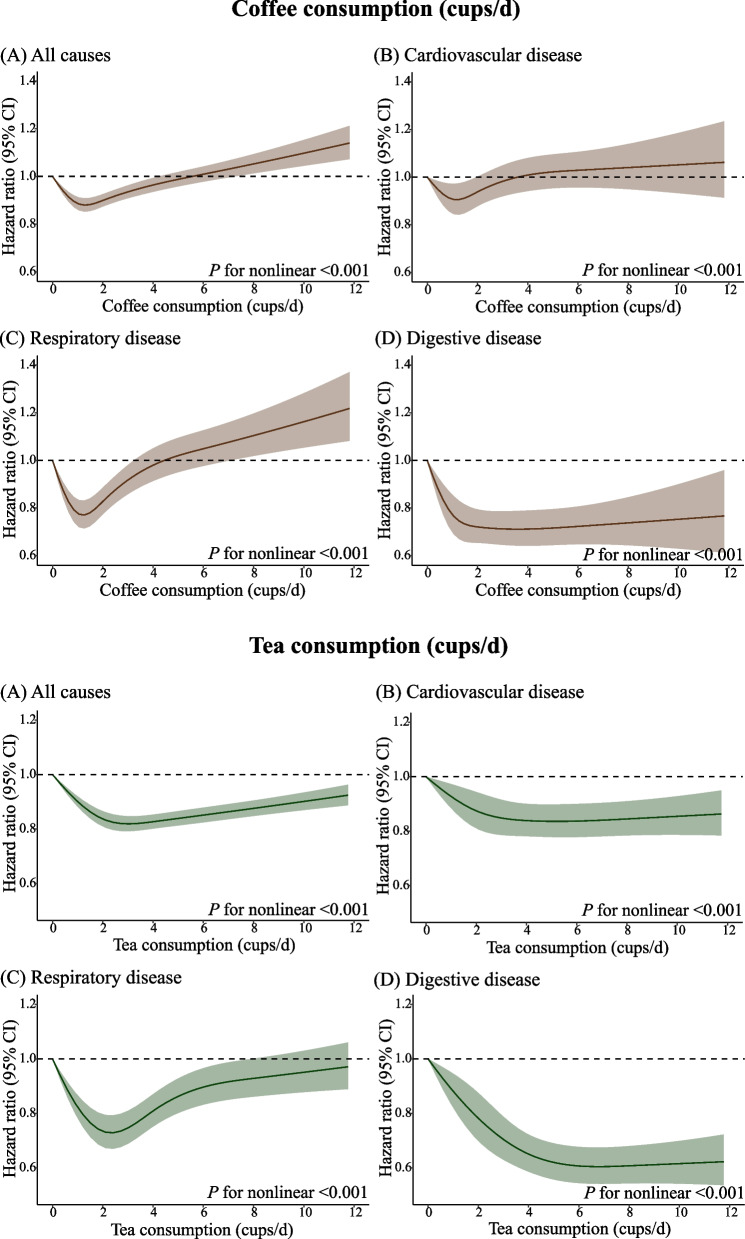
Restricted spline curve for associations of coffee and tea consumption with all-cause and cause-specific mortality. Models were adjusted for sex, age, ethnicity, education levels, BMI, smoking status, alcohol intake frequency, physical activity, dietary pattern, general health status, hypertension, diabetes, and depression. Coffee and tea consumption were mutually adjusted

In Fig. 1, the dose-response relationship of separate coffee and tea consumption with all-cause, CVD, respiratory disease, and digestive disease mortality were significantly nonlinear (all *P*-nonlinear < 0.001). A multivariable-adjusted model showed J-shaped associations between separate coffee consumption with all-cause, CVD, and respiratory disease mortality and separate tea consumption with respiratory disease mortality. The reverse J-shaped associations were observed for separate tea consumption with all-cause and CVD mortality. The threshold consumption for the lowest mortality risk was about 1 cup/day of coffee and 3 cups/day of tea. Particularly, reverse J-shaped associations were observed between separate coffee and tea consumption with digestive disease mortality, and participants who consumed about 5 cups/day of coffee and 6 cups/day of tea showed the lowest risk.

### Separate coffee and tea consumption with mortality

To quantify the magnitude of relationships between coffee and tea consumption with mortality, we categorized coffee (none, < 1–2, 3–4, and ≥ 5 cups/day) and tea (none, < 1–1, 2–4, and ≥ 5 cups/day) consumption groups according to prior validated results of restricted cubic spline analyses in the present study. After adjusting for potential confounders, < 1–2 cups/day of coffee was inversely associated with a lower risk of health outcomes: 0.91 (0.88–0.93) for all-cause, 0.94 (0.88–1.00) for CVD, and 0.84 (0.78–0.89) for respiratory disease mortality (Table 2). Participants who drank 2–4 cups/day of tea had a lower risk for all-cause (HR, 0.86; 95% CI: 0.83–0.88), CVD (HR, 0.88; 95% CI: 0.81–0.94), and respiratory disease mortality (HR, 0.80; 95% CI: 0.74–0.87). Additionally, we found that ≥ 5 cups/day of coffee (HR, 0.67; 95% CI: 0.59–0.77) and ≥ 5 cups/day of tea (HR, 0.65; 95% CI: 0.58–0.73) showed the lowest risk for digestive disease mortality, which was similar to the results of restricted cubic splines.

**Table 2:** HRs and 95% CIs of individual coffee and tea consumption for all-cause and cause-specific mortality

Cause of death	All participants	Coffee consumption (cups/day)				Tea consumption (cups/day)			
		None (*N* = 110,646)	< 1–2 (*N* = 229,498)	3–4 (*N* = 102,694)	≥ 5 (*N* = 55,320)	None (*N* = 73,223)	< 1–1 (*N* = 57,438)	2–4 (*N* = 217,454)	≥ 5 (*N* = 150,043)
All causes									
No. of deaths (%)	34,699	7963 (22.95)	15,023 (43.30)	7124 (20.53)	4589 (13.23)	5681 (16.37)	3744 (10.79)	14,206 (40.94)	11,068 (31.90)
Model 1^a^		1.00 (Ref.)	0.80 (0.78–0.83)	0.84 (0.81–0.86)	1.03 (0.99–1.07)	1.00 (Ref.)	0.84 (0.80–0.87)	0.77 (0.75–0.80)	0.86 (0.83–0.89)
Model 2^b^		1.00 (Ref.)	0.91 (0.88–0.93)	0.92 (0.89–0.95)	0.97 (0.93–1.01)	1.00 (Ref.)	0.92 (0.88–0.96)	0.86 (0.83–0.88)	0.87 (0.84–0.90)
Cardiovascular disease									
No. of deaths (%)	6663	1528 (22.93)	2854 (42.83)	1366 (20.50)	915 (13.73)	1106 (16.60)	731 (10.97)	2748 (41.24)	2078 (31.19)
Model 1^a^		1.00 (Ref.)	0.79 (0.74–0.84)	0.81 (0.75–0.88)	1.02 (0.94–1.12)	1.00 (Ref.)	0.84 (0.76–0.92)	0.77 (0.71–0.83)	0.82 (0.75–0.88)
Model 2^b^		1.00 (Ref.)	0.94 (0.88–1.00)	0.96 (0.89–1.04)	1.04 (0.95–1.14)	1.00 (Ref.)	0.94 (0.85–1.03)	0.88 (0.81–0.94)	0.87 (0.80–0.94)
Respiratory disease									
No. of deaths (%)	6018	1532 (25.46)	2354 (39.12)	1173 (19.49)	959 (15.94)	1078 (17.91)	549 (9.12)	2247 (37.34)	2144 (35.63)
Model 1^a^		1.00 (Ref.)	0.67 (0.63–0.71)	0.75 (0.69–0.81)	1.16 (1.06–1.26)	1.00 (Ref.)	0.68 (0.61–0.75)	0.69 (0.64–0.75)	0.92 (0.85–0.99)
Model 2^b^		1.00 (Ref.)	0.84 (0.78–0.89)	0.90 (0.83–0.98)	1.06 (0.97–1.16)	1.00 (Ref.)	0.80 (0.72–0.88)	0.80 (0.74–0.87)	0.91 (0.84–0.99)
Digestive disease									
No. of deaths (%)	2864	768 (26.82)	1189 (41.52)	544 (18.99)	363 (12.67)	533 (18.61)	351 (12.26)	1155 (40.33)	825 (28.81)
Model 1^a^		1.00 (Ref.)	0.66 (0.60–0.72)	0.60 (0.54–0.68)	0.72 (0.63–0.82)	1.00 (Ref.)	0.84 (0.73–0.96)	0.65 (0.58–0.72)	0.61 (0.55–0.69)
Model 2^b^		1.00 (Ref.)	0.78 (0.71–0.86)	0.69 (0.62–0.78)	0.67 (0.59–0.77)	1.00 (Ref.)	0.95 (0.83–1.09)	0.76 (0.68–0.85)	0.65 (0.58–0.73)

*Abbreviation*s: *HR* hazard ratio; *CI* confidence interval

^a^Adjusted for sex and age

^b^Adjusted for sex, age, ethnicity, education levels, BMI, smoking status, alcohol intake frequency, physical activity, dietary pattern, general health status, hypertension, diabetes, and depression. Coffee and tea consumption were mutually adjusted

### Effects of combined coffee and tea consumption on mortality

**Fig. 2:**
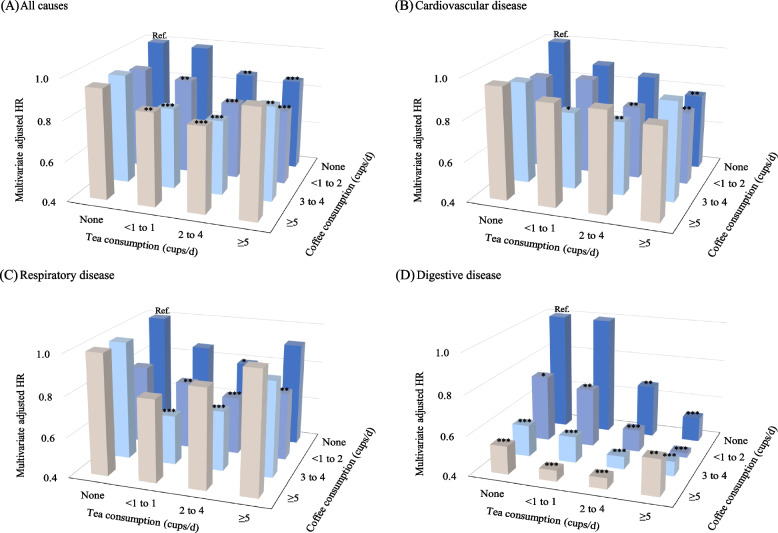
Combined consumption of coffee and tea with all-cause and cause-specific mortality. Models were adjusted for sex, age, ethnicity, education levels, BMI, smoking status, alcohol intake frequency, physical activity, dietary pattern, general health status, hypertension, diabetes, and depression. *P < 0.05, **P < 0.01, ***P < 0.001

We further explored the relationship of the combined associations of coffee and tea consumption with mortality. We found significant interactions between coffee and tea consumption with all-cause and digestive disease mortality. (*P* for interaction < 0.01). As shown in Fig. 2 and Additional file 1: Table S2, for individuals with low coffee consumption, the increasing tea consumption was almost linearly associated with all-cause mortality, whereas for those with high coffee consumption, the relationship was U-shaped. However, the increasing coffee consumption showed a U-shaped relationship with all-cause mortality for those with low or high tea consumption. Specifically, compared with those who did not drink coffee and tea, participants who drank both < 1–2 cups of coffee and 2–4 cups of tea per day were associated with a 22% lower risk for all-cause (HR, 0.78; 95% CI: 0.73–0.85), 24% lower risk for CVD (HR, 0.76; 95% CI: 0.64–0.91), and 31% lower risk for respiratory disease mortality (HR, 0.69; 95% CI: 0.57–0.83). Nevertheless, the lowest HR (95% CI) of drinking both < 1–2 cup/day of coffee and ≥ 5 cups/day of tea for digestive disease mortality was 0.42 (0.34–0.53).

### Subgroup analyses

**Fig. 3:**
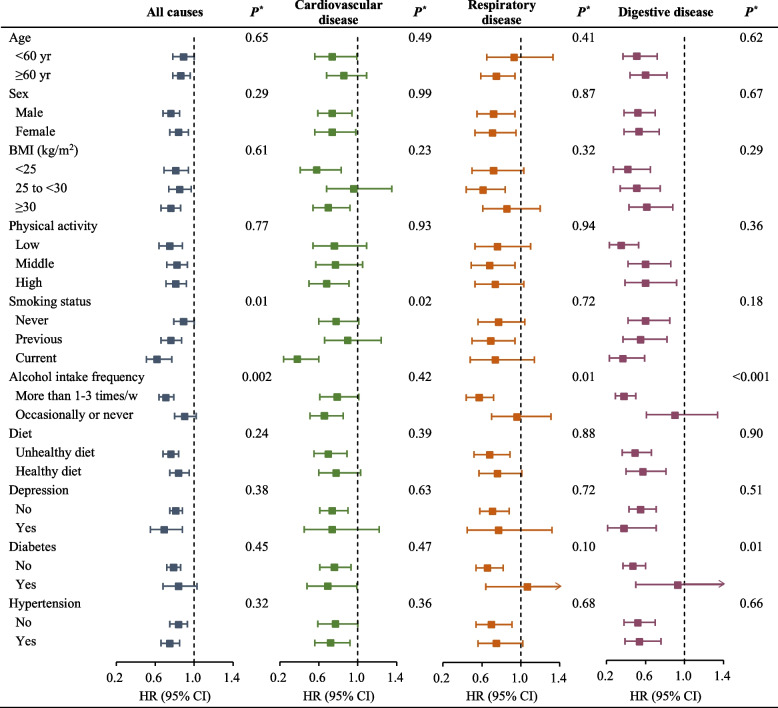
Multivariable HRs and 95% CIs of combined effect of coffee and tea consumption for all-cause and cause-specific mortality in subgroups. Multivariable HRs for all-cause mortality among study participants who consumed combined < 1–2 cups/day of coffee and 2–4 cups/day of tea versus those who drink neither coffee nor tea were adjusted for sex, age, ethnicity, education levels, BMI, smoking status, alcohol intake frequency, physical activity, dietary pattern, general health status, hypertension, diabetes, and depression. *P for interaction between baseline characteristics and combined consumption of coffee and tea with all-cause or cause-specific mortality. The risk estimates for other categories of combined consumption of coffee and tea are shown in Table S3-S12 in the Supplementary Appendix

Associations of separate coffee and tea consumption with all-cause and cause-specific mortality did not differ by sex, age, BMI, physical activity, smoking status, alcohol intake frequency, dietary pattern, hypertension, diabetes, and depression (Additional file 1: Tables S3-S12). Figure 3 shows associations between the combined consumption of coffee and tea with mortality by subgroup analyses. Multivariable hazard ratios were calculated for total and cause-specific mortality among study participants who consumed < 1–2 cups/day of coffee and 2–4 cups/day of tea compared to those who drank neither coffee nor tea. In the analyses stratified by potential confounding factors for death, the inverse associations between combined consumption of coffee and tea with mortality were presented in all subgroups. In terms of strata of smoking status, the association was stronger among current smoking participants than among never and previous smoking participants (*P* for interaction = 0.01). Similarly, a multiplicative interaction was also found in the stratification of alcohol intake frequency (*P* for interaction = 0.002), and the HR of the group with low alcohol intake frequency was lower than that of those with high alcohol intake frequency. The details of the combined effects of coffee and tea consumption for each group were shown in Additional file 1: Tables S3-S12, and the results were not much altered with stratification by these factors.

### Sensitivity analyses

The results were not apparently altered in the following sensitivity analyses. After excluding those who died within 3 years, the association between separate and combined coffee and tea consumption and all-cause and cause-specific mortality remained significant (Additional file 1: Table S13). When the analyses were performed following the exclusion of all participants with CVD and cancer at baseline, the results were not altered (Additional file 1: Table S14). When the analyses were conducted after adjusting for pack-years categories of cigarette smoking, the results were basically unchanged (Additional file 1: Table S15). The results also did not change materially when the main analyses were conducted without adjusting for baseline depression (Additional file 1: Table S16). Moreover, the significant interaction between coffee and tea consumption on all-cause and digestive disease mortality remained in the above analyses (*P* for interaction < 0.05). Furthermore, the findings were similar when we repeated the analysis using data that excluded the missing values of covariates rather than multiple imputations (Additional file 1: Table S17).

## Discussion

This large prospective study investigated the associations between separate and combined consumption of coffee and tea and mortality. We found that the association between separate coffee consumption and the risk of all-cause mortality was J-shaped, whereas that of separate tea consumption was reverse J-shaped. As compared with participants who drank neither coffee nor tea, those who drank < 1–2 cups/day of coffee and 2–4 cups/day of tea had a 22% lower risk of mortality. Inverse associations were also observed for CVD, respiratory disease, and digestive disease mortality. Moreover, similar associations were sustained among all subgroups.

In recent decades, many prospective epidemiological studies have investigated the associations between separate coffee and tea consumption with major causes of death, but the observed J-shaped and U-shaped associations remain controversial [27, 28]. A prospective study conducted in the Health Examinees study revealed that drinking > 3 cups/day of coffee was associated with a 21% lower risk for all-cause mortality [29]. Another study included 100,902 general Chinese adults showed an inverse association between habitual tea drinkers and all-cause mortality compared with never or non-habitual tea drinkers [30]. Our findings are also consistent with previous studies on CVD, respiratory disease, and digestive disease mortality [31–33]. The J-shaped association observed in our study was inconsistent with a previous study that reported excess coffee drinking (≥ 8 cups/day) was still inversely associated with all-cause mortality in the UK Biobank [10]. The heterogeneous findings may be due to differences in study design, population inclusion, longer follow-up, and increased all-cause death cases. The underlying mechanisms hypothesized to explain the lack of further risk reduction for heavy coffee and tea drinking on mortality risk may be that caffeine, overlapping bioactive compounds to coffee and tea, could increase heart rate, blood pressure, and induce insulin resistance [34]. Tannins in coffee and tea decrease calcium and iron absorption, and coffee contains diterpenes that increase cholesterol levels in the blood [28, 35]. Therefore, although excessive coffee and tea consumption were not associated with an increased risk of digestive disease mortality, the higher risk of all-cause and other specific causes of mortality makes it imperative to support the incorporation of moderate coffee and tea consumption into the dietary pattern.

Beyond separate associations of coffee and tea consumption with mortality, the potential joint effect of these two key beverages remains largely unknown. Only one study conducted in the Fukuoka Diabetes Registry including 4923 patients (2790 men and 2133 women) with type 2 diabetes followed for 5.3 years revealed that the combined consumption of ≥ 2 cups/day of coffee and ≥ 4 cups/day of green tea had 63% lowest risk for all-cause mortality [14]. Although this analysis considered the combined association and interaction effect of coffee and tea consumption, some limitations merit consideration. First, the population size and follow-up time were limited, which is prone to reverse causality. Second, the classification of coffee and tea consumption in this study was crude. We made a detailed classification of coffee and tea consumption, which was able to accurately assess the relationship with mortality. Third, this study focused on patients with type 2 diabetes, and the dietary guidance is difficult to generalize to the general population. In our study, we found that the combination of < 1–2 cups/day of coffee and 2–4 cups/day of tea had a 22% lower risk for all-cause, 24% lower risk for CVD, and 31% lower risk for respiratory disease mortality. Nevertheless, drinking both < 1–2 cup/day of coffee and ≥ 5 cups/day of tea had a 58% lower risk for digestive disease mortality. Future studies are needed to validate these results.

Several biological mechanisms have been hypothesized to explain why combined coffee and tea consumption is inversely associated with the risk of mortality. First, coffee and tea, two popular refreshing beverages, have overlapping bioactive components such as caffeine and chlorogenic acid. They play a crucial role in antioxidants, anti-inflammation, lowering blood pressure, insulin resistance, and improving endothelial function [5, 36, 37]. The pathogenesis of most chronic diseases involved these mechanisms. Second, some evidence that coffee consumption was associated with inverse diseases, and decaffeinated consumption did not change these associations [38]. It is therefore reasonable to think that different bioactive substances in coffee and tea also play a protective role. In addition to caffeine and chlorogenic acid, coffee also contains other compounds, such as trigonelline, melanoidins, magnesium, and cafestol, which have known anti-oxidant properties and may decrease mortality [39]. Tea contains unique compounds, such as epicatechin, catechin, epigallocatechin-3-gallate (EGCG), and other flavonoids. These can alleviate atherosclerosis, scavenge oxygen-free radicals, and attenuate inflammation [40–42]. Third, many studies have indicated that coffee and tea consumption are associated with a lower risk of biological indicators related to mortality, such as blood pressure, glucose, and triglycerides [43–45]. Therefore, biological indicators could be controlled to reduce the risk of mortality, but their role in these associations needs to be further studied. Fourth, several interpretations could explain the interaction of smoking and drinking. Smoking stimulates the metabolism and clearance of caffeine, and thereby smokers have higher tolerance to caffeine [46]. In other words, when consuming the same amount of caffeine, nonsmokers have two to three times higher plasma caffeine concentration than smokers [47]. For alcohol drinking, caffeine blocks the alcohol adenosine A1 receptor, which antagonizes the adverse effects of alcohol [48]. Finally, the protective effect of coffee and tea consumption was significant for digestive disease mortality. This is because the ingredients have the function of secretion of bile acids, microbiome composition, and fecal output to improve the digestive system environment [49]. Future mechanistic work on the joint association between coffee and tea consumption is needed before more robust conclusions can be made.

The strengths of this study included the population-based study design with a 15-year follow-up. To the best of our knowledge, the present study is the first to assess the combined associations of coffee and tea consumption with mortality in the general population. More importantly, instead of assuming linearity between them, we explored the dose-response relationship using restricted cubic splines. We also examined the joint effect and interaction between coffee and tea consumption, fully adjusting for confounding factors. Despite the strengths of the present study, several potential limitations should be mentioned. First, data on coffee and tea consumption were collected through self-reported questionnaires, and potential response bias cannot be ruled out. Also, certain baseline information is likely to vary with time during a rather long follow-up period. Second, our conclusions may be affected by reverse causation, although results were similar after excluding the first 3 years of follow-up. Third, as with any observational study design, residual confounding is possible, especially for smoking. Although we have adjusted for pack-years categories of cigarette smoking, we could not avoid the possibility of residual effects due to unmeasured confounders between former and current smokers. Fourth, due to the large sample size in our study, the significance we found such as the interaction between coffee and tea consumption may be attained by chance, although similar results were obtained in multiple sensitivity analyses. Finally, the majority of participants are of European descent in the UK biobank, and they are more health-conscious than the general population [50]. Caution is therefore needed when generalizing our findings to other ethnicities.

## Conclusions

Our results provided evidence that the association between separate coffee consumption and the risk of all-cause mortality was J-shaped, whereas that of separate tea consumption was reverse J-shaped. Our findings do not support that drinking excess coffee is inversely associated with mortality. Our study underscores that it is of vital significance to integrate < 1–2 cups/day of coffee and 2–4 cups/day of tea into diet patterns and health management programs if observed associations are causal. Further studies are warranted to confirm our findings and clarify the potential mechanism by which combined coffee and tea consumption are associated with mortality.

## Supplementary Information

**Additional file 1: Table S1.** Diet component definitions used in the UK Biobank study. **Table S2.** Multivariable HRs and 95% CIs of combined effect of coffee and tea consumption on total and cause-specific mortality. **Table S3.** Multivariable HRs and 95% CIs of separate and combined effect of coffee and tea consumption on total and cause-specific mortality by sex. **Table S4.** Multivariable HRs and 95% CIs of separate and combined effect of coffee and tea consumption on total and cause-specific mortality by age group (<60 and ≥60 years). **Table S5.** Multivariable HRs and 95% CIs of separate and combined effect of coffee and tea consumption on total and cause-specific mortality by BMI. **Table S6.** Multivariable HRs and 95% CIs of separate and combined effect of coffee and tea consumption on total and cause-specific mortality by physical activity. **Table S7.** Multivariable HRs and 95% CIs of separate and combined effect of coffee and tea consumption on total and cause-specific mortality by smoking status. **Table S8.** Multivariable HRs and 95% CIs of separate and combined effect of coffee and tea consumption on total and cause-specific mortality by alcohol intake frequency. **Table S9.** Multivariable HRs and 95% CIs of separate and combined effect of coffee and tea consumption on total and cause-specific mortality by diet pattern. **Table S10.** Multivariable HRs and 95% CIs of separate and combined effect of coffee and tea consumption on total and cause-specific mortality by depression. **Table S11.** Multivariable HRs and 95% CIs of separate and combined effect of coffee and tea consumption on total and cause-specific mortality by diabetes. **Table S12.** Multivariable HRs and 95% CIs of separate and combined effect of coffee and tea consumption on total and cause-specific mortality by hypertension. **Table S13.** Multivariable HRs and 95% CIs of separate and combined effect of coffee and tea consumption on total and cause-specific mortality after exclusion of first three years of follow-up. **Table S14.** Multivariable HRs and 95% CIs of separate and combined effect of coffee and tea consumption on total and cause-specific mortality after exclusion patients with prevalent CVD and cancer at baseline. **Table S15.** Multivariable HRs and 95% CIs of separate and combined effect of coffee and tea consumption on total and cause-specific mortality adjusted for pack-years categories of cigarette smoking at baseline. **Table S16.** Multivariable HRs and 95% CIs of separate and combined effect of coffee and tea consumption on total and cause-specific mortality unadjusted for depression at baseline. **Table S17.** Multivariable HRs and 95% CIs of separate and combined effect of coffee and tea consumption on total and cause-specific mortality using unimputed data. **Figure S1.** Flowchart for the selection of the analyzed study sample from the UK Biobank Study. **Figure S2.** Directed acyclic graph (DAG) derived from previous literature and expert knowledge. **Figure S3.** The distribution of combination of coffee and tea consumption.

## Data Availability

The data that support the findings of this study are available from UK Biobank (https://www.ukbiobank.ac.uk/), but restrictions apply to the availability of these data, which were used under license for the current study, and so are not publicly available. Data are however available from the authors upon reasonable request and with permission of UK Biobank.

## Abbreviations

BMI: Body mass index
CI: Confidence intervals
CVD: Cardiovascular disease
DAG: Directed acyclic graph
HR: Hazard ratio
ICD: International Classification of Diseases
IPAQ: The International Physical Activity Questionnaire
IQR: Interquartile range
LRT: Likelihood ratio test
MICE: Multiple imputation using chained equations.
SD: Standard deviation
