# Supplementary material for: Consumption of coffee and tea with all-cause and cause-specific mortality: a prospective cohort study
Source: BMC Med. 2022 Nov 18;20:449. doi: 10.1186/s12916-022-02636-2 (PMC9673438; doi:10.1186/s12916-022-02636-2)
Supplement: Supplementary file 1 — Additional file 1: Table S1. Diet component definitions used in the UK Biobank study. Table S2. Multivariable HRs and 95% CIs of combined effect of coffee and tea consumption on total and cause-specific mortality. Table S3. Multivariable HRs and 95% CIs of separate and combined effect of coffee and tea consumption on total and cause-specific mortality by sex. Table S4. Multivariable HRs and 95% CIs of separate and combined effect of coffee and tea consumption on total and cause-specific mortality by age group (<60 and ≥60 years). Table S5. Multivariable HRs and 95% CIs of separate and combined effect of coffee and tea consumption on total and cause-specific mortality by BMI. Table S6. Multivariable HRs and 95% CIs of separate and combined effect of coffee and tea consumption on total and cause-specific mortality by physical activity. Table S7. Multivariable HRs and 95% CIs of separate and combined effect of coffee and tea consumption on total and cause-specific mortality by smoking status. Table S8. Multivariable HRs and 95% CIs of separate and combined effect of coffee and tea consumption on total and cause-specific mortality by alcohol intake frequency. Table S9. Multivariable HRs and 95% CIs of separate and combined effect of coffee and tea consumption on total and cause-specific mortality by diet pattern. Table S10. Multivariable HRs and 95% CIs of separate and combined effect of coffee and tea consumption on total and cause-specific mortality by depression. Table S11. Multivariable HRs and 95% CIs of separate and combined effect of coffee and tea consumption on total and cause-specific mortality by diabetes. Table S12. Multivariable HRs and 95% CIs of separate and combined effect of coffee and tea consumption on total and cause-specific mortality by hypertension. Table S13. Multivariable HRs and 95% CIs of separate and combined effect of coffee and tea consumption on total and cause-specific mortality after exclusion of first three years of follow-up. Table S14 [file 12916_2022_2636_MOESM1_ESM.docx]

**Additional File 1**

**Table S1.** Diet component definitions used in the UK Biobank study

**Table S2.** Multivariable HRs and 95% CIs of combined effect of coffee and tea consumption on total and cause-specific mortality

**Table S3.** Multivariable HRs and 95% CIs of separate and combined effect of coffee and tea consumption on total and cause-specific mortality by sex

**Table S4.** Multivariable HRs and 95% CIs of separate and combined effect of coffee and tea consumption on total and cause-specific mortality by age group (<60 and ≥60 years)

**Table S5.** Multivariable HRs and 95% CIs of separate and combined effect of coffee and tea consumption on total and cause-specific mortality by BMI

**Table S6.** Multivariable HRs and 95% CIs of separate and combined effect of coffee and tea consumption on total and cause-specific mortality by physical activity

**Table S7.** Multivariable HRs and 95% CIs of separate and combined effect of coffee and tea consumption on total and cause-specific mortality by smoking status

**Table S8.** Multivariable HRs and 95% CIs of separate and combined effect of coffee and tea consumption on total and cause-specific mortality by alcohol intake frequency

**Table S9.** Multivariable HRs and 95% CIs of separate and combined effect of coffee and tea consumption on total and cause-specific mortality by diet pattern

**Table S10.** Multivariable HRs and 95% CIs of separate and combined effect of coffee and tea consumption on total and cause-specific mortality by depression

**Table S11.** Multivariable HRs and 95% CIs of separate and combined effect of coffee and tea consumption on total and cause-specific mortality by diabetes.

**Table S12.** Multivariable HRs and 95% CIs of separate and combined effect of coffee and tea consumption on total and cause-specific mortality by hypertension

**Table S13.** Multivariable HRs and 95% CIs of separate and combined effect of coffee and tea consumption on total and cause-specific mortality after exclusion of first three years of follow-up

**Table S14.** Multivariable HRs and 95% CIs of separate and combined effect of coffee and tea consumption on total and cause-specific mortality after exclusion patients with prevalent CVD and cancer at baseline

**Table S15.** Multivariable HRs and 95% CIs of separate and combined effect of coffee and tea consumption on total and cause-specific mortality adjusted for pack-years categories of cigarette smoking at baseline

**Table S16.** Multivariable HRs and 95% CIs of separate and combined effect of coffee and tea consumption on total and cause-specific mortality unadjusted for depression at baseline

**Table S17.** Multivariable HRs and 95% CIs of separate and combined effect of coffee and tea consumption on total and cause-specific mortality using unimputed data

**Fig. S1.** Flowchart for the selection of the analyzed study sample from the UK Biobank Study

**Fig. S2.** Directed acyclic graph (DAG) derived from previous literature and expert knowledge

**Fig. S3.** The distribution of combination of coffee and tea consumption

**Table S1.** Diet component definitions used in the UK Biobank study

| Source and definition of healthy diet | Self-reported UK Biobank field code |
| --- | --- |
| Fruits: ≥ 3 servings/day | 1309, 1319 |
| Vegetables: ≥ 3 servings/day | 1289, 1299 |
| Fish: ≥2 servings/week | 1329, 1339 |
| Processed meats: ≤1 serving/week | 1349 |
| Unprocessed red meats: ≤ 1.5 servings/week | 1369, 1379, 1389, |
| Whole grains: ≥ 3servings/day | 1438, 1448, 1458, 1468 |
| Ref.ined grains: ≤1.5 servings/day | 1438, 1448, 1458, 1468 |

A healthy diet was based on adherence to at least four of seven commonly eaten food groups following recommendations on dietary priorities for cardiometabolic health.

**Table S2.** Multivariable HRs and 95% CIs of combined effect of coffee and tea consumption on total and cause-specific mortality

| Group | | All-causes | P value | Cardiovascular disease | P value | Respiratory disease | P value | Digestive disease | P value |
| --- | --- | --- | --- | --- | --- | --- | --- | --- | --- |
| **Coffee** | **Tea** |  |  |  |  |  |  |  |  |
| 0 | 0 | 1 (Ref.) | Ref. | 1 (Ref.) | Ref. | 1 (Ref.) | Ref. | 1 (Ref.) | Ref. |
| 0 | <1-1 | 0.99 (0.87,1.12) | 0.882 | 0.90 (0.68,1.19) | 0.454 | 0.86 (0.63,1.18) | 0.353 | 1.00 (0.71,1.40) | 0.993 |
| 0 | 2-4 | 0.87 (0.80,0.94) | 0.001 | 0.86 (0.71,1.03) | 0.100 | 0.80 (0.65,0.97) | 0.026 | 0.67 (0.53,0.84) | 0.001 |
| 0 | ≥5 | 0.86 (0.79,0.93) | <0.001 | 0.78 (0.65,0.94) | 0.007 | 0.92 (0.76,1.11) | 0.380 | 0.52 (0.42,0.66) | <0.001 |
| <1-2 | 0 | 0.91 (0.83,1.00) | 0.052 | 0.87 (0.70,1.08) | 0.202 | 0.80 (0.63,1.00) | 0.054 | 0.74 (0.57,0.96) | 0.025 |
| <1-2 | <1-1 | 0.88 (0.80,0.96) | 0.005 | 0.88 (0.72,1.08) | 0.221 | 0.74 (0.59,0.93) | 0.008 | 0.70 (0.54,0.90) | 0.006 |
| <1-2 | 2-4 | 0.78 (0.73,0.85) | <0.001 | 0.76 (0.64,0.91) | 0.003 | 0.69 (0.57,0.83) | <0.001 | 0.51 (0.41,0.63) | <0.001 |
| <1-2 | ≥5 | 0.77 (0.72,0.84) | <0.001 | 0.77 (0.64,0.92) | 0.003 | 0.74 (0.61,0.90) | 0.002 | 0.42 (0.34,0.53) | <0.001 |
| 3-4 | 0 | 0.95 (0.87,1.03) | 0.207 | 0.91 (0.75,1.11) | 0.346 | 0.99 (0.80,1.22) | 0.911 | 0.56 (0.43,0.73) | <0.001 |
| 3-4 | <1-1 | 0.80 (0.73,0.88) | <0.001 | 0.78 (0.63,0.97) | 0.026 | 0.65 (0.51,0.82) | <0.001 | 0.53 (0.40,0.71) | <0.001 |
| 3-4 | 2-4 | 0.77 (0.71,0.84) | <0.001 | 0.77 (0.64,0.92) | 0.005 | 0.70 (0.57,0.85) | <0.001 | 0.46 (0.37,0.59) | <0.001 |
| 3-4 | ≥5 | 0.87 (0.79,0.95) | 0.003 | 0.89 (0.72,1.10) | 0.295 | 0.88 (0.70,1.09) | 0.242 | 0.47 (0.35,0.64) | <0.001 |
| ≥5 | 0 | 0.95 (0.87,1.03) | 0.218 | 0.96 (0.79,1.16) | 0.643 | 1.03 (0.84,1.26) | 0.781 | 0.54 (0.42,0.70) | <0.001 |
| ≥5 | <1-1 | 0.86 (0.78,0.95) | 0.004 | 0.90 (0.72,1.14) | 0.384 | 0.81 (0.63,1.04) | 0.093 | 0.46 (0.33,0.63) | <0.001 |
| ≥5 | 2-4 | 0.82 (0.75,0.91) | <0.001 | 0.90 (0.73,1.11) | 0.327 | 0.89 (0.71,1.12) | 0.313 | 0.45 (0.34,0.61) | <0.001 |
| ≥5 | ≥5 | 0.94 (0.85,1.04) | 0.215 | 0.85 (0.67,1.08) | 0.184 | 1.06 (0.84,1.33) | 0.634 | 0.58 (0.43,0.80) | 0.001 |

Abbreviation: HR, hazard ratio; CI, confidence interval.

Models were adjusted for sex, age, ethnicity, education levels, BMI, smoking status, alcohol intake frequency, physical activity, dietary pattern, general health status, hypertension, diabetes, and depression.

**Table S3.** Multivariable HRs and 95% CIs of separate and combined effect of coffee and tea consumption on total and cause-specific mortality by sex

| Group | | Male | | | |  | Female | | | |
| --- | --- | --- | --- | --- | --- | --- | --- | --- | --- | --- |
|  |  | All causes | Cardiovascular disease | Respiratory disease | Digestive disease |  | All causes | Cardiovascular disease | Respiratory disease | Digestive disease |
| **Coffee** |  |  |  |  |  |  |  |  |  |  |
| 0 |  | 1.00 (Ref.) | 1.00 (Ref.) | 1.00 (Ref.) | 1.00 (Ref.) |  | 1.00 (Ref.) | 1.00 (Ref.) | 1.00 (Ref.) | 1.00 (Ref.) |
| <1-2 |  | 0.90(0.87,0.93) | 0.96(0.89,1.04) | 0.83(0.77,0.90) | 0.76(0.67,0.86) |  | 0.93(0.89,0.97) | 0.90(0.80,1.00) | 0.85(0.76,0.95) | 0.82(0.70,0.95) |
| 3-4 |  | 0.91(0.87,0.95) | 0.97(0.88,1.06) | 0.90(0.82,1.00) | 0.67(0.58,0.77) |  | 0.95(0.90,1.00) | 0.93(0.80,1.06) | 0.91(0.79,1.04) | 0.73(0.60,0.88) |
| ≥5 |  | 0.97(0.92,1.02) | 1.04(0.94,1.16) | 1.14(1.02,1.27) | 0.65(0.55,0.77) |  | 0.96(0.90,1.03) | 1.02(0.87,1.20) | 0.92(0.79,1.07) | 0.70(0.55,0.88) |
| **Tea** |  |  |  |  |  |  |  |  |  |  |
| 0 |  | 1.00 (Ref.) | 1.00 (Ref.) | 1.00 (Ref.) | 1.00 (Ref.) |  | 1.00 (Ref.) | 1.00 (Ref.) | 1.00 (Ref.) | 1.00 (Ref.) |
| <1-1 |  | 0.90(0.85,0.95) | 0.91(0.81,1.02) | 0.80(0.70,0.91) | 1.00(0.85,1.19) |  | 0.96(0.90,1.02) | 1.01(0.85,1.20) | 0.80(0.68,0.96) | 0.85(0.67,1.08) |
| 2-4 |  | 0.85(0.82,0.89) | 0.87(0.79,0.95) | 0.80(0.73,0.89) | 0.75(0.66,0.87) |  | 0.86(0.82,0.91) | 0.89(0.78,1.02) | 0.80(0.71,0.91) | 0.78(0.65,0.93) |
| ≥5 |  | 0.86(0.82,0.90) | 0.88(0.80,0.97) | 0.94(0.85,1.04) | 0.63(0.54,0.73) |  | 0.88(0.83,0.93) | 0.84(0.73,0.97) | 0.86(0.75,0.99) | 0.69(0.56,0.83) |
| **Coffee** | **Tea** |  |  |  |  |  |  |  |  |  |
| 0 | 0 | 1.00 (Ref.) | 1.00 (Ref.) | 1.00 (Ref.) | 1.00 (Ref.) |  | 1.00 (Ref.) | 1.00 (Ref.) | 1.00 (Ref.) | 1.00 (Ref.) |
| 0 | <1-1 | 0.98(0.83,1.16) | 0.89(0.62,1.27) | 0.86(0.57,1.29) | 1.24(0.82,1.89) |  | 0.99(0.82,1.19) | 0.93(0.58,1.49) | 0.91(0.56,1.47) | 0.62(0.33,1.17) |
| 0 | 2-4 | 0.83(0.74,0.93) | 0.82(0.65,1.05) | 0.81(0.62,1.06) | 0.69(0.51,0.95) |  | 0.91(0.81,1.02) | 0.93(0.69,1.24) | 0.78(0.58,1.05) | 0.64(0.45,0.91) |
| 0 | ≥5 | 0.81(0.73,0.91) | 0.78(0.62,0.99) | 0.93(0.71,1.20) | 0.54(0.40,0.73) |  | 0.90(0.80,1.01) | 0.78(0.58,1.04) | 0.92(0.70,1.21) | 0.52(0.37,0.72) |
| <1-2 | 0 | 0.86(0.76,0.98) | 0.88(0.67,1.16) | 0.81(0.59,1.10) | 0.82(0.58,1.16) |  | 0.97(0.84,1.10) | 0.85(0.60,1.20) | 0.79(0.56,1.12) | 0.63(0.42,0.96) |
| <1-2 | <1-1 | 0.82(0.73,0.93) | 0.82(0.63,1.06) | 0.72(0.53,0.98) | 0.76(0.54,1.06) |  | 0.95(0.83,1.08) | 1.03(0.75,1.43) | 0.79(0.56,1.10) | 0.59(0.39,0.90) |
| <1-2 | 2-4 | 0.75(0.67,0.83) | 0.77(0.61,0.96) | 0.68(0.53,0.88) | 0.51(0.38,0.68) |  | 0.82(0.74,0.92) | 0.76(0.57,1.00) | 0.72(0.54,0.94) | 0.52(0.38,0.72) |
| <1-2 | ≥5 | 0.72(0.65,0.81) | 0.77(0.62,0.97) | 0.75(0.58,0.97) | 0.41(0.31,0.56) |  | 0.83(0.75,0.93) | 0.74(0.56,0.98) | 0.74(0.56,0.97) | 0.44(0.32,0.62) |
| 3-4 | 0 | 0.88(0.78,0.99) | 0.93(0.72,1.19) | 0.92(0.69,1.22) | 0.57(0.40,0.80) |  | 1.03(0.91,1.16) | 0.87(0.63,1.20) | 1.10(0.82,1.50) | 0.55(0.37,0.81) |
| 3-4 | <1-1 | 0.74(0.65,0.84) | 0.73(0.56,0.96) | 0.69(0.50,0.95) | 0.55(0.38,0.79) |  | 0.90(0.78,1.03) | 0.92(0.65,1.30) | 0.57(0.39,0.85) | 0.50(0.32,0.8) |
| 3-4 | 2-4 | 0.73(0.65,0.82) | 0.77(0.60,0.97) | 0.69(0.52,0.90) | 0.48(0.35,0.66) |  | 0.82(0.73,0.92) | 0.76(0.56,1.02) | 0.74(0.55,0.99) | 0.44(0.30,0.64) |
| 3-4 | ≥5 | 0.84(0.74,0.95) | 0.86(0.66,1.12) | 0.95(0.71,1.28) | 0.46(0.31,0.67) |  | 0.89(0.77,1.02) | 0.97(0.68,1.38) | 0.73(0.51,1.05) | 0.52(0.33,0.83) |
| ≥5 | 0 | 0.93(0.82,1.04) | 0.92(0.72,1.17) | 1.13(0.86,1.49) | 0.55(0.39,0.76) |  | 0.95(0.84,1.08) | 1.04(0.76,1.41) | 0.87(0.64,1.18) | 0.54(0.37,0.79) |
| ≥5 | <1-1 | 0.79(0.69,0.91) | 0.96(0.73,1.27) | 0.81(0.58,1.12) | 0.46(0.30,0.70) |  | 0.95(0.82,1.11) | 0.72(0.47,1.11) | 0.82(0.56,1.20) | 0.45(0.26,0.77) |
| ≥5 | 2-4 | 0.77(0.68,0.88) | 0.82(0.63,1.08) | 0.92(0.68,1.24) | 0.48(0.33,0.70) |  | 0.90(0.78,1.04) | 1.12(0.78,1.61) | 0.85(0.59,1.22) | 0.39(0.23,0.67) |
| ≥5 | ≥5 | 0.91(0.80,1.04) | 0.88(0.66,1.17) | 1.15(0.85,1.56) | 0.59(0.40,0.87) |  | 0.94(0.79,1.10) | 0.71(0.45,1.13) | 0.88(0.60,1.30) | 0.58(0.34,1.00) |

Abbreviation: HR, hazard ratio; CI, confidence interval.

Models were adjusted for age, ethnicity, education levels, BMI, smoking status, alcohol intake frequency, physical activity, dietary pattern, general health status, hypertension, diabetes, and depression. Coffee and tea consumption were mutually adjusted.

**Table S4.** Multivariable HRs and 95% CIs of separate and combined effect of coffee and tea consumption on total and cause-specific mortality by age group (<60 and ≥60 years)

| Group | | <60 | | | |  | ≥60 | | | |
| --- | --- | --- | --- | --- | --- | --- | --- | --- | --- | --- |
|  |  | All causes | Cardiovascular disease | Respiratory disease | Digestive disease |  | All causes | Cardiovascular disease | Respiratory disease | Digestive disease |
| **Coffee** |  |  |  |  |  |  |  |  |  |  |
| 0 |  | 1.00 (Ref.) | 1.00 (Ref.) | 1.00 (Ref.) | 1.00 (Ref.) |  | 1.00 (Ref.) | 1.00 (Ref.) | 1.00 (Ref.) | 1.00 (Ref.) |
| <1-2 |  | 0.96(0.91,1.01) | 0.91(0.81,1.03) | 0.97(0.85,1.12) | 0.80(0.68,0.93) |  | 0.93(0.90,0.96) | 0.98(0.90,1.05) | 0.84(0.77,0.90) | 0.80(0.71,0.90) |
| 3-4 |  | 0.97(0.91,1.03) | 1.00(0.86,1.15) | 1.09(0.92,1.28) | 0.63(0.51,0.77) |  | 0.94(0.90,0.98) | 0.97(0.88,1.06) | 0.89(0.81,0.97) | 0.75(0.65,0.86) |
| ≥5 |  | 1.00(0.93,1.07) | 1.01(0.86,1.18) | 1.27(1.07,1.50) | 0.69(0.56,0.86) |  | 0.97(0.92,1.02) | 1.05(0.94,1.17) | 1.00(0.90,1.11) | 0.66(0.55,0.79) |
| **Tea** |  |  |  |  |  |  |  |  |  |  |
| 0 |  | 1.00 (Ref.) | 1.00 (Ref.) | 1.00 (Ref.) | 1.00 (Ref.) |  | 1.00 (Ref.) | 1.00 (Ref.) | 1.00 (Ref.) | 1.00 (Ref.) |
| <1-1 |  | 0.92(0.86,0.99) | 0.97(0.82,1.15) | 0.78(0.63,0.95) | 0.74(0.59,0.93) |  | 0.93(0.89,0.98) | 0.93(0.83,1.05) | 0.81(0.72,0.91) | 1.11(0.94,1.32) |
| 2-4 |  | 0.91(0.85,0.96) | 0.91(0.79,1.04) | 0.82(0.70,0.96) | 0.70(0.59,0.83) |  | 0.87(0.84,0.91) | 0.89(0.82,0.98) | 0.83(0.76,0.91) | 0.85(0.74,0.98) |
| ≥5 |  | 0.92(0.87,0.98) | 0.95(0.82,1.10) | 0.99(0.85,1.16) | 0.55(0.46,0.67) |  | 0.89(0.85,0.92) | 0.87(0.79,0.96) | 0.92(0.83,1.01) | 0.75(0.64,0.87) |
| **Coffee** | **Tea** |  |  |  |  |  |  |  |  |  |
| 0 | 0 | 1.00 (Ref.) | 1.00 (Ref.) | 1.00 (Ref.) | 1.00 (Ref.) |  | 1.00 (Ref.) | 1.00 (Ref.) | 1.00 (Ref.) | 1.00 (Ref.) |
| 0 | <1-1 | 0.99(0.82,1.21) | 0.93(0.60,1.45) | 0.98(0.57,1.69) | 0.82(0.49,1.38) |  | 1.01(0.85,1.19) | 0.92(0.64,1.34) | 0.84(0.57,1.22) | 1.21(0.77,1.90) |
| 0 | 2-4 | 0.95(0.83,1.08) | 0.96(0.72,1.28) | 0.87(0.60,1.25) | 0.75(0.54,1.04) |  | 0.90(0.81,1.01) | 0.91(0.72,1.16) | 0.83(0.65,1.05) | 0.70(0.51,0.96) |
| 0 | ≥5 | 0.91(0.80,1.03) | 0.81(0.61,1.08) | 1.11(0.79,1.55) | 0.50(0.36,0.70) |  | 0.91(0.82,1.01) | 0.86(0.68,1.09) | 0.92(0.73,1.16) | 0.60(0.44,0.82) |
| <1-2 | 0 | 0.92(0.79,1.07) | 0.63(0.43,0.92) | 0.87(0.56,1.35) | 0.93(0.64,1.36) |  | 0.97(0.86,1.09) | 1.05(0.81,1.38) | 0.82(0.62,1.07) | 0.69(0.48,1.00) |
| <1-2 | <1-1 | 0.94(0.81,1.08) | 0.84(0.60,1.18) | 0.79(0.51,1.21) | 0.66(0.45,0.98) |  | 0.93(0.83,1.04) | 0.99(0.76,1.28) | 0.78(0.60,1.01) | 0.79(0.56,1.12) |
| <1-2 | 2-4 | 0.89(0.79,1.01) | 0.79(0.60,1.04) | 0.94(0.67,1.31) | 0.52(0.38,0.72) |  | 0.84(0.76,0.93) | 0.86(0.68,1.08) | 0.70(0.56,0.88) | 0.57(0.42,0.78) |
| <1-2 | ≥5 | 0.88(0.78,0.99) | 0.87(0.66,1.15) | 1.04(0.74,1.46) | 0.40(0.29,0.57) |  | 0.83(0.75,0.92) | 0.83(0.66,1.05) | 0.74(0.59,0.93) | 0.49(0.36,0.66) |
| 3-4 | 0 | 1.02(0.89,1.17) | 1.07(0.78,1.46) | 1.32(0.91,1.93) | 0.57(0.38,0.84) |  | 1.00(0.89,1.11) | 0.94(0.73,1.21) | 0.97(0.76,1.25) | 0.61(0.43,0.87) |
| 3-4 | <1-1 | 0.86(0.73,1.00) | 0.82(0.57,1.17) | 0.89(0.57,1.40) | 0.47(0.29,0.74) |  | 0.86(0.77,0.97) | 0.86(0.66,1.13) | 0.64(0.48,0.85) | 0.63(0.43,0.91) |
| 3-4 | 2-4 | 0.85(0.74,0.97) | 0.75(0.55,1.01) | 0.86(0.59,1.25) | 0.40(0.27,0.59) |  | 0.83(0.74,0.92) | 0.86(0.68,1.10) | 0.72(0.56,0.91) | 0.55(0.40,0.76) |
| 3-4 | ≥5 | 1.02(0.87,1.18) | 1.07(0.77,1.51) | 1.25(0.83,1.87) | 0.48(0.30,0.77) |  | 0.90(0.80,1.02) | 0.93(0.71,1.22) | 0.85(0.65,1.11) | 0.53(0.36,0.78) |
| ≥5 | 0 | 1.02(0.89,1.16) | 0.96(0.71,1.30) | 1.42(1.00,2.02) | 0.65(0.45,0.92) |  | 0.98(0.87,1.09) | 1.03(0.80,1.33) | 0.96(0.75,1.22) | 0.52(0.36,0.74) |
| ≥5 | <1-1 | 0.9(0.76,1.06) | 1.01(0.71,1.45) | 1.08(0.70,1.68) | 0.35(0.20,0.60) |  | 0.90(0.79,1.03) | 0.90(0.67,1.22) | 0.76(0.56,1.03) | 0.57(0.37,0.87) |
| ≥5 | 2-4 | 0.88(0.75,1.02) | 0.85(0.60,1.21) | 1.15(0.77,1.73) | 0.54(0.35,0.83) |  | 0.87(0.77,0.98) | 1.01(0.77,1.33) | 0.87(0.66,1.14) | 0.43(0.28,0.66) |
| ≥5 | ≥5 | 1(0.85,1.17) | 0.80(0.54,1.18) | 1.24(0.81,1.89) | 0.56(0.35,0.89) |  | 0.99(0.87,1.13) | 0.95(0.71,1.29) | 1.06(0.80,1.40) | 0.64(0.42,0.98) |

Abbreviation: HR, hazard ratio; CI, confidence interval.

Models were adjusted for sex, ethnicity, education levels, BMI, smoking status, alcohol intake frequency, physical activity, dietary pattern, general health status, hypertension, diabetes, and depression. Coffee and tea consumption were mutually adjusted.

**Table S5.** Multivariable HRs and 95% CIs of separate and combined effect of coffee and tea consumption on total and cause-specific mortality by BMI

| Group | | BMI <25 | | | |  | BMI 25 to <30 | | | |  | BMI ≥30 | | | |
| --- | --- | --- | --- | --- | --- | --- | --- | --- | --- | --- | --- | --- | --- | --- | --- |
|  |  | All causes | Cardiovascular disease | Respiratory disease | Digestive disease |  | All causes | Cardiovascular disease | Respiratory disease | Digestive disease |  | All causes | Cardiovascular disease | Respiratory disease | Digestive disease |
| **Coffee** |  |  |  |  |  |  |  |  |  |  |  |  |  |  |  |
| 0 |  | 1.00 (Ref.) | 1.00 (Ref.) | 1.00 (Ref.) | 1.00 (Ref.) |  | 1.00 (Ref.) | 1.00 (Ref.) | 1.00 (Ref.) | 1.00 (Ref.) |  | 1.00 (Ref.) | 1.00 (Ref.) | 1.00 (Ref.) | 1.00 (Ref.) |
| <1-2 |  | 0.92(0.87,0.97) | 0.90(0.78,1.03) | 0.81(0.71,0.91) | 0.76(0.63,0.92) |  | 0.87(0.84,0.91) | 0.95(0.86,1.05) | 0.85(0.76,0.95) | 0.77(0.66,0.90) |  | 0.96(0.91,1.01) | 0.96(0.87,1.07) | 0.86(0.77,0.96) | 0.82(0.70,0.95) |
| 3-4 |  | 0.95(0.89,1.02) | 0.89(0.75,1.06) | 1.01(0.87,1.17) | 0.64(0.50,0.81) |  | 0.90(0.85,0.94) | 0.93(0.82,1.05) | 0.88(0.77,1.00) | 0.73(0.60,0.88) |  | 0.93(0.87,0.99) | 1.03(0.91,1.17) | 0.86(0.75,0.99) | 0.71(0.58,0.85) |
| ≥5 |  | 1.01(0.93,1.09) | 1.12(0.92,1.37) | 1.06(0.90,1.26) | 0.70(0.54,0.91) |  | 0.97(0.91,1.03) | 1.04(0.90,1.20) | 1.19(1.03,1.38) | 0.61(0.48,0.77) |  | 0.92(0.86,0.99) | 1.01(0.87,1.16) | 0.92(0.78,1.07) | 0.70(0.56,0.86) |
| **Tea** |  |  |  |  |  |  |  |  |  |  |  |  |  |  |  |
| 0 |  | 1.00 (Ref.) | 1.00 (Ref.) | 1.00 (Ref.) | 1.00 (Ref.) |  | 1.00 (Ref.) | 1.00 (Ref.) | 1.00 (Ref.) | 1.00 (Ref.) |  | 1.00 (Ref.) | 1.00 (Ref.) | 1.00 (Ref.) | 1.00 (Ref.) |
| <1-1 |  | 0.96(0.88,1.04) | 0.88(0.70,1.09) | 0.87(0.71,1.05) | 1.03(0.79,1.33) |  | 0.93(0.87,1.00) | 1.05(0.91,1.22) | 0.78(0.66,0.92) | 1.00(0.79,1.28) |  | 0.88(0.82,0.95) | 0.87(0.75,1.01) | 0.77(0.64,0.92) | 0.87(0.70,1.08) |
| 2-4 |  | 0.83(0.78,0.88) | 0.92(0.78,1.10) | 0.82(0.70,0.95) | 0.66(0.53,0.82) |  | 0.89(0.84,0.94) | 0.90(0.80,1.02) | 0.79(0.69,0.90) | 0.87(0.72,1.05) |  | 0.84(0.80,0.89) | 0.84(0.75,0.95) | 0.82(0.72,0.94) | 0.76(0.64,0.90) |
| ≥5 |  | 0.85(0.79,0.90) | 0.90(0.75,1.07) | 0.94(0.80,1.09) | 0.50(0.39,0.63) |  | 0.89(0.84,0.94) | 0.86(0.76,0.98) | 0.93(0.81,1.06) | 0.71(0.58,0.87) |  | 0.87(0.82,0.92) | 0.87(0.77,0.98) | 0.87(0.76,1.00) | 0.72(0.60,0.86) |
| **Coffee** | **Tea** |  |  |  |  |  |  |  |  |  |  |  |  |  |  |
| 0 | 0 | 1.00 (Ref.) | 1.00 (Ref.) | 1.00 (Ref.) | 1.00 (Ref.) |  | 1.00 (Ref.) | 1.00 (Ref.) | 1.00 (Ref.) | 1.00 (Ref.) |  | 1.00 (Ref.) | 1.00 (Ref.) | 1.00 (Ref.) | 1.00 (Ref.) |
| 0 | <1-1 | 1.25(1.00,1.56) | 0.67(0.36,1.23) | 1.16(0.68,1.98) | 1.42(0.80,2.54) |  | 0.97(0.78,1.20) | 1.10(0.67,1.81) | 0.50(0.28,0.92) | 0.69(0.36,1.31) |  | 0.85(0.69,1.05) | 0.93(0.61,1.43) | 1.07(0.65,1.77) | 1.00(0.57,1.76) |
| 0 | 2-4 | 0.88(0.75,1.03) | 0.75(0.52,1.08) | 0.84(0.59,1.21) | 0.67(0.44,1.04) |  | 0.97(0.84,1.12) | 1.10(0.78,1.56) | 0.67(0.48,0.93) | 0.63(0.43,0.94) |  | 0.77(0.68,0.89) | 0.78(0.59,1.03) | 0.92(0.65,1.29) | 0.70(0.48,1.01) |
| 0 | ≥5 | 0.88(0.76,1.02) | 0.68(0.47,0.96) | 0.94(0.67,1.33) | 0.37(0.24,0.58) |  | 0.93(0.81,1.07) | 0.95(0.68,1.34) | 0.77(0.56,1.06) | 0.51(0.35,0.76) |  | 0.78(0.68,0.88) | 0.75(0.58,0.99) | 1.08(0.78,1.49) | 0.65(0.45,0.93) |
| <1-2 | 0 | 0.98(0.82,1.17) | 0.80(0.52,1.23) | 0.70(0.45,1.09) | 0.68(0.40,1.16) |  | 0.92(0.79,1.09) | 0.99(0.66,1.47) | 0.73(0.49,1.07) | 0.62(0.38,0.99) |  | 0.86(0.75,1.00) | 0.87(0.63,1.18) | 0.93(0.64,1.37) | 0.89(0.59,1.33) |
| <1-2 | <1-1 | 0.93(0.79,1.10) | 0.65(0.43,0.98) | 0.77(0.51,1.15) | 0.75(0.46,1.20) |  | 0.94(0.81,1.10) | 1.23(0.85,1.77) | 0.63(0.44,0.92) | 0.67(0.44,1.04) |  | 0.80(0.69,0.93) | 0.81(0.59,1.10) | 0.84(0.57,1.24) | 0.67(0.44,1.03) |
| <1-2 | 2-4 | 0.80(0.69,0.93) | 0.64(0.46,0.90) | 0.67(0.47,0.94) | 0.44(0.29,0.67) |  | 0.83(0.73,0.95) | 0.94(0.68,1.32) | 0.58(0.42,0.79) | 0.49(0.34,0.71) |  | 0.76(0.67,0.86) | 0.75(0.58,0.98) | 0.87(0.63,1.20) | 0.60(0.42,0.84) |
| <1-2 | ≥5 | 0.82(0.71,0.96) | 0.65(0.46,0.91) | 0.80(0.57,1.12) | 0.38(0.24,0.58) |  | 0.80(0.70,0.92) | 0.96(0.69,1.35) | 0.62(0.45,0.85) | 0.40(0.27,0.58) |  | 0.73(0.65,0.83) | 0.73(0.56,0.96) | 0.84(0.60,1.16) | 0.49(0.34,0.70) |
| 3-4 | 0 | 1.08(0.91,1.28) | 0.69(0.45,1.05) | 1.11(0.76,1.63) | 0.62(0.37,1.02) |  | 0.97(0.84,1.13) | 1.11(0.77,1.60) | 0.76(0.53,1.08) | 0.51(0.33,0.81) |  | 0.85(0.74,0.98) | 0.92(0.69,1.23) | 1.16(0.82,1.65) | 0.58(0.38,0.87) |
| 3-4 | <1-1 | 0.94(0.79,1.12) | 0.57(0.36,0.90) | 0.91(0.60,1.39) | 0.55(0.32,0.95) |  | 0.84(0.72,0.99) | 1.07(0.73,1.57) | 0.55(0.37,0.82) | 0.42(0.26,0.70) |  | 0.70(0.60,0.82) | 0.73(0.53,1.02) | 0.55(0.36,0.86) | 0.65(0.42,1.01) |
| 3-4 | 2-4 | 0.81(0.69,0.94) | 0.66(0.45,0.95) | 0.80(0.56,1.15) | 0.37(0.23,0.59) |  | 0.83(0.72,0.96) | 0.94(0.66,1.33) | 0.56(0.40,0.78) | 0.52(0.35,0.77) |  | 0.70(0.61,0.80) | 0.75(0.57,0.99) | 0.79(0.56,1.12) | 0.47(0.32,0.69) |
| 3-4 | ≥5 | 0.86(0.72,1.03) | 0.74(0.48,1.13) | 0.97(0.65,1.44) | 0.35(0.19,0.64) |  | 0.91(0.78,1.06) | 0.91(0.61,1.34) | 0.73(0.50,1.05) | 0.42(0.25,0.69) |  | 0.85(0.73,0.99) | 1.05(0.77,1.44) | 0.97(0.66,1.44) | 0.63(0.40,1.00) |
| ≥5 | 0 | 1.02(0.87,1.20) | 0.78(0.53,1.16) | 1.11(0.77,1.59) | 0.61(0.38,0.97) |  | 1.03(0.89,1.20) | 1.23(0.86,1.76) | 0.93(0.66,1.30) | 0.39(0.25,0.61) |  | 0.83(0.72,0.95) | 0.90(0.68,1.2) | 1.06(0.75,1.49) | 0.63(0.42,0.93) |
| ≥5 | <1-1 | 0.94(0.77,1.14) | 0.83(0.51,1.34) | 0.71(0.44,1.15) | 0.48(0.26,0.90) |  | 0.91(0.77,1.08) | 1.19(0.79,1.78) | 0.73(0.48,1.09) | 0.52(0.30,0.88) |  | 0.77(0.65,0.91) | 0.78(0.55,1.11) | 0.96(0.63,1.46) | 0.36(0.20,0.65) |
| ≥5 | 2-4 | 0.99(0.83,1.18) | 0.97(0.64,1.47) | 1.05(0.71,1.57) | 0.48(0.28,0.84) |  | 0.88(0.75,1.03) | 1.10(0.74,1.61) | 0.74(0.51,1.07) | 0.37(0.22,0.63) |  | 0.66(0.56,0.78) | 0.76(0.54,1.06) | 0.90(0.61,1.35) | 0.51(0.31,0.82) |
| ≥5 | ≥5 | 0.93(0.76,1.13) | 0.83(0.52,1.34) | 0.96(0.62,1.48) | 0.50(0.27,0.92) |  | 1.03(0.87,1.22) | 0.93(0.60,1.42) | 1.22(0.84,1.76) | 0.47(0.27,0.83) |  | 0.85(0.72,1.01) | 0.86(0.60,1.23) | 0.86(0.56,1.32) | 0.75(0.46,1.22) |

Abbreviation: HR, hazard ratio; CI, confidence interval.

Models were adjusted for sex, age, ethnicity, education levels, smoking status, alcohol intake frequency, physical activity, dietary pattern, general health status, hypertension, diabetes, and depression. Coffee and tea consumption were mutually adjusted.

**Table S6.** Multivariable HRs and 95% CIs of separate and combined effect of coffee and tea consumption on total and cause-specific mortality by physical activity

| Group | | Low | | | |  | Middle | | | |  | High | | | |
| --- | --- | --- | --- | --- | --- | --- | --- | --- | --- | --- | --- | --- | --- | --- | --- |
|  |  | All causes | Cardiovascular disease | Respiratory disease | Digestive disease |  | All causes | Cardiovascular disease | Respiratory disease | Digestive disease |  | All causes | Cardiovascular disease | Respiratory disease | Digestive disease |
| **Coffee** |  |  |  |  |  |  |  |  |  |  |  |  |  |  |  |
| 0 |  | 1.00 (Ref.) | 1.00 (Ref.) | 1.00 (Ref.) | 1.00 (Ref.) |  | 1.00 (Ref.) | 1.00 (Ref.) | 1.00 (Ref.) | 1.00 (Ref.) |  | 1.00 (Ref.) | 1.00 (Ref.) | 1.00 (Ref.) | 1.00 (Ref.) |
| <1-2 |  | 0.92(0.87,0.98) | 0.92(0.81,1.05) | 0.90(0.79,1.02) | 0.75(0.63,0.89) |  | 0.92(0.88,0.96) | 0.93(0.84,1.03) | 0.80(0.72,0.89) | 0.80(0.69,0.93) |  | 0.89(0.85,0.93) | 0.97(0.87,1.08) | 0.83(0.74,0.93) | 0.79(0.66,0.93) |
| 3-4 |  | 0.91(0.85,0.97) | 0.92(0.79,1.07) | 0.96(0.82,1.11) | 0.60(0.48,0.76) |  | 0.91(0.86,0.96) | 0.97(0.85,1.10) | 0.89(0.78,1.01) | 0.65(0.54,0.79) |  | 0.94(0.89,1.00) | 0.98(0.86,1.12) | 0.88(0.77,1.02) | 0.83(0.68,1.02) |
| ≥5 |  | 0.90(0.83,0.97) | 1.01(0.85,1.21) | 0.98(0.83,1.16) | 0.50(0.39,0.66) |  | 0.98(0.92,1.04) | 1.02(0.89,1.18) | 1.10(0.96,1.27) | 0.60(0.48,0.75) |  | 1.01(0.95,1.08) | 1.09(0.93,1.26) | 1.08(0.93,1.27) | 0.95(0.76,1.20) |
| **Tea** |  |  |  |  |  |  |  |  |  |  |  |  |  |  |  |
| 0 |  | 1.00 (Ref.) | 1.00 (Ref.) | 1.00 (Ref.) | 1.00 (Ref.) |  | 1.00 (Ref.) | 1.00 (Ref.) | 1.00 (Ref.) | 1.00 (Ref.) |  | 1.00 (Ref.) | 1.00 (Ref.) | 1.00 (Ref.) | 1.00 (Ref.) |
| <1-1 |  | 0.97(0.89,1.05) | 0.99(0.83,1.20) | 0.82(0.68,0.99) | 0.96(0.75,1.23) |  | 0.94(0.88,1.01) | 0.92(0.79,1.07) | 0.79(0.67,0.94) | 0.96(0.78,1.20) |  | 0.87(0.81,0.93) | 0.92(0.78,1.09) | 0.78(0.65,0.95) | 0.93(0.73,1.20) |
| 2-4 |  | 0.83(0.78,0.89) | 1.01(0.87,1.17) | 0.77(0.66,0.88) | 0.61(0.50,0.75) |  | 0.86(0.82,0.91) | 0.81(0.72,0.91) | 0.83(0.74,0.95) | 0.81(0.68,0.96) |  | 0.87(0.82,0.92) | 0.87(0.77,0.99) | 0.82(0.71,0.94) | 0.85(0.70,1.04) |
| ≥5 |  | 0.83(0.77,0.89) | 0.88(0.75,1.03) | 0.83(0.72,0.97) | 0.57(0.46,0.71) |  | 0.87(0.82,0.92) | 0.82(0.72,0.93) | 0.91(0.80,1.04) | 0.61(0.50,0.73) |  | 0.90(0.85,0.95) | 0.91(0.80,1.04) | 0.99(0.86,1.14) | 0.79(0.64,0.97) |
| **Coffee** | **Tea** |  |  |  |  |  |  |  |  |  |  |  |  |  |  |
| 0 | 0 | 1.00 (Ref.) | 1.00 (Ref.) | 1.00 (Ref.) | 1.00 (Ref.) |  | 1.00 (Ref.) | 1.00 (Ref.) | 1.00 (Ref.) | 1.00 (Ref.) |  | 1.00 (Ref.) | 1.00 (Ref.) | 1.00 (Ref.) | 1.00 (Ref.) |
| 0 | <1-1 | 0.94(0.74,1.19) | 0.71(0.41,1.23) | 1.09(0.66,1.81) | 0.72(0.38,1.35) |  | 1.05(0.86,1.29) | 1.11(0.71,1.75) | 0.66(0.37,1.17) | 1.36(0.82,2.24) |  | 0.97(0.78,1.21) | 0.88(0.54,1.42) | 0.90(0.51,1.58) | 0.87(0.43,1.75) |
| 0 | 2-4 | 0.82(0.70,0.97) | 0.89(0.63,1.27) | 0.77(0.53,1.12) | 0.56(0.37,0.84) |  | 0.91(0.80,1.04) | 0.94(0.69,1.29) | 0.90(0.65,1.24) | 0.70(0.48,1.01) |  | 0.86(0.75,0.98) | 0.75(0.56,1.02) | 0.73(0.51,1.03) | 0.76(0.50,1.17) |
| 0 | ≥5 | 0.79(0.68,0.92) | 0.76(0.53,1.08) | 0.85(0.60,1.21) | 0.46(0.31,0.69) |  | 0.87(0.76,0.99) | 0.82(0.61,1.11) | 0.94(0.69,1.29) | 0.52(0.36,0.75) |  | 0.90(0.79,1.03) | 0.76(0.57,1.02) | 0.99(0.71,1.36) | 0.60(0.40,0.92) |
| <1-2 | 0 | 0.93(0.78,1.11) | 0.82(0.54,1.23) | 0.88(0.58,1.32) | 0.62(0.39,0.98) |  | 0.96(0.83,1.12) | 1.00(0.71,1.41) | 0.82(0.56,1.20) | 0.87(0.58,1.31) |  | 0.83(0.71,0.98) | 0.79(0.55,1.13) | 0.70(0.46,1.07) | 0.70(0.41,1.17) |
| <1-2 | <1-1 | 0.87(0.73,1.03) | 0.87(0.59,1.29) | 0.74(0.49,1.12) | 0.78(0.50,1.21) |  | 0.92(0.79,1.06) | 0.89(0.64,1.25) | 0.74(0.51,1.07) | 0.68(0.45,1.03) |  | 0.85(0.73,0.99) | 0.89(0.64,1.24) | 0.76(0.51,1.12) | 0.62(0.38,1.02) |
| <1-2 | 2-4 | 0.75(0.65,0.88) | 0.8(0.57,1.12) | 0.74(0.52,1.05) | 0.38(0.26,0.56) |  | 0.80(0.71,0.91) | 0.79(0.59,1.06) | 0.67(0.49,0.92) | 0.57(0.40,0.80) |  | 0.79(0.70,0.90) | 0.73(0.55,0.97) | 0.70(0.50,0.96) | 0.58(0.39,0.87) |
| <1-2 | ≥5 | 0.73(0.63,0.85) | 0.71(0.50,1.00) | 0.75(0.52,1.06) | 0.35(0.23,0.53) |  | 0.82(0.72,0.93) | 0.85(0.63,1.14) | 0.77(0.56,1.05) | 0.44(0.31,0.63) |  | 0.76(0.67,0.87) | 0.73(0.55,0.98) | 0.73(0.53,1.02) | 0.48(0.32,0.73) |
| 3-4 | 0 | 0.89(0.75,1.05) | 0.83(0.56,1.22) | 1.10(0.75,1.61) | 0.50(0.32,0.79) |  | 0.93(0.81,1.07) | 0.97(0.70,1.35) | 0.96(0.68,1.35) | 0.52(0.34,0.79) |  | 1.01(0.88,1.17) | 0.92(0.66,1.27) | 0.95(0.66,1.37) | 0.69(0.43,1.10) |
| 3-4 | <1-1 | 0.84(0.70,1.01) | 0.77(0.50,1.17) | 0.72(0.46,1.12) | 0.47(0.28,0.79) |  | 0.85(0.73,0.98) | 0.98(0.70,1.38) | 0.67(0.46,0.99) | 0.51(0.33,0.81) |  | 0.75(0.63,0.87) | 0.61(0.42,0.89) | 0.58(0.38,0.89) | 0.64(0.38,1.07) |
| 3-4 | 2-4 | 0.72(0.61,0.84) | 0.78(0.54,1.12) | 0.70(0.48,1.02) | 0.33(0.21,0.52) |  | 0.82(0.72,0.93) | 0.83(0.61,1.13) | 0.78(0.56,1.07) | 0.54(0.38,0.79) |  | 0.77(0.67,0.88) | 0.71(0.52,0.96) | 0.64(0.45,0.90) | 0.50(0.32,0.78) |
| 3-4 | ≥5 | 0.79(0.66,0.96) | 0.81(0.53,1.25) | 0.87(0.57,1.33) | 0.33(0.18,0.60) |  | 0.83(0.71,0.97) | 0.91(0.64,1.29) | 0.83(0.58,1.20) | 0.33(0.19,0.55) |  | 0.96(0.83,1.12) | 0.94(0.67,1.31) | 0.96(0.66,1.39) | 0.82(0.51,1.33) |
| ≥5 | 0 | 0.84(0.71,0.99) | 0.82(0.57,1.20) | 0.96(0.66,1.39) | 0.44(0.28,0.68) |  | 1.01(0.88,1.16) | 1.17(0.85,1.61) | 1.13(0.81,1.57) | 0.50(0.33,0.75) |  | 0.98(0.85,1.13) | 0.86(0.62,1.18) | 1.01(0.71,1.43) | 0.73(0.47,1.15) |
| ≥5 | <1-1 | 0.86(0.70,1.04) | 0.90(0.58,1.39) | 0.86(0.55,1.35) | 0.26(0.13,0.51) |  | 0.91(0.78,1.08) | 0.95(0.65,1.39) | 0.91(0.61,1.35) | 0.46(0.27,0.78) |  | 0.80(0.68,0.96) | 0.87(0.59,1.27) | 0.67(0.43,1.06) | 0.72(0.41,1.25) |
| ≥5 | 2-4 | 0.72(0.60,0.88) | 1.06(0.71,1.59) | 0.77(0.50,1.18) | 0.24(0.12,0.45) |  | 0.83(0.71,0.97) | 0.80(0.55,1.15) | 0.99(0.69,1.43) | 0.45(0.28,0.74) |  | 0.90(0.76,1.05) | 0.89(0.63,1.27) | 0.92(0.63,1.36) | 0.71(0.43,1.18) |
| ≥5 | ≥5 | 0.79(0.64,0.98) | 0.70(0.43,1.13) | 0.98(0.63,1.52) | 0.49(0.27,0.87) |  | 0.95(0.81,1.13) | 0.88(0.60,1.31) | 1.02(0.70,1.51) | 0.52(0.31,0.88) |  | 1.02(0.87,1.21) | 0.94(0.65,1.37) | 1.18(0.80,1.74) | 0.77(0.45,1.31) |

Abbreviation: HR, hazard ratio; CI, confidence interval.

Models were adjusted for sex, age, ethnicity, education levels, BMI, smoking status, alcohol intake frequency, dietary pattern, general health status, hypertension, diabetes, and depression. Coffee and tea consumption were mutually adjusted.

**Table S7.** Multivariable HRs and 95% CIs of separate and combined effect of coffee and tea consumption on total and cause-specific mortality by smoking status

| Group | | Never | | | |  | Previous | | | |  | Current | | | |
| --- | --- | --- | --- | --- | --- | --- | --- | --- | --- | --- | --- | --- | --- | --- | --- |
|  |  | All causes | Cardiovascular disease | Respiratory disease | Digestive disease |  | All causes | Cardiovascular disease | Respiratory disease | Digestive disease |  | All causes | Cardiovascular disease | Respiratory disease | Digestive disease |
| **Coffee** |  |  |  |  |  |  |  |  |  |  |  |  |  |  |  |
| 0 |  | 1.00 (Ref.) | 1.00 (Ref.) | 1.00 (Ref.) | 1.00 (Ref.) |  | 1.00 (Ref.) | 1.00 (Ref.) | 1.00 (Ref.) | 1.00 (Ref.) |  | 1.00 (Ref.) | 1.00 (Ref.) | 1.00 (Ref.) | 1.00 (Ref.) |
| <1-2 |  | 0.94(0.90,0.98) | 0.94(0.85,1.04) | 0.91(0.80,1.03) | 0.75(0.65,0.88) |  | 0.91(0.87,0.95) | 1.00(0.90,1.10) | 0.80(0.73,0.89) | 0.79(0.68,0.91) |  | 0.86(0.81,0.92) | 0.82(0.70,0.96) | 0.85(0.74,0.97) | 0.85(0.69,1.04) |
| 3-4 |  | 0.94(0.89,1.00) | 1.00(0.88,1.13) | 0.87(0.74,1.02) | 0.66(0.55,0.81) |  | 0.92(0.87,0.97) | 0.97(0.86,1.09) | 0.89(0.79,1.01) | 0.73(0.61,0.88) |  | 0.90(0.83,0.98) | 0.89(0.74,1.06) | 0.97(0.83,1.13) | 0.68(0.54,0.87) |
| ≥5 |  | 1.00(0.93,1.07) | 1.15(0.99,1.34) | 1.13(0.94,1.38) | 0.65(0.50,0.83) |  | 0.95(0.89,1.01) | 0.98(0.85,1.13) | 1.03(0.89,1.18) | 0.69(0.55,0.86) |  | 0.95(0.88,1.03) | 0.99(0.82,1.19) | 1.07(0.92,1.25) | 0.67(0.52,0.86) |
| **Tea** |  |  |  |  |  |  |  |  |  |  |  |  |  |  |  |
| 0 |  | 1.00 (Ref.) | 1.00 (Ref.) | 1.00 (Ref.) | 1.00 (Ref.) |  | 1.00 (Ref.) | 1.00 (Ref.) | 1.00 (Ref.) | 1.00 (Ref.) |  | 1.00 (Ref.) | 1.00 (Ref.) | 1.00 (Ref.) | 1.00 (Ref.) |
| <1-1 |  | 0.98(0.92,1.05) | 0.91(0.79,1.06) | 0.96(0.79,1.18) | 0.96(0.76,1.22) |  | 0.92(0.86,0.98) | 0.96(0.83,1.12) | 0.75(0.64,0.88) | 1.03(0.82,1.28) |  | 0.84(0.76,0.92) | 0.92(0.74,1.13) | 0.72(0.59,0.88) | 0.85(0.65,1.11) |
| 2-4 |  | 0.90(0.85,0.95) | 0.85(0.76,0.96) | 0.90(0.77,1.06) | 0.83(0.69,1.00) |  | 0.85(0.81,0.90) | 0.93(0.83,1.05) | 0.75(0.67,0.85) | 0.78(0.65,0.93) |  | 0.78(0.73,0.84) | 0.78(0.66,0.93) | 0.80(0.69,0.93) | 0.66(0.53,0.82) |
| ≥5 |  | 0.87(0.82,0.93) | 0.81(0.71,0.92) | 0.9(0.76,1.06) | 0.68(0.56,0.83) |  | 0.86(0.81,0.91) | 0.91(0.80,1.03) | 0.88(0.78,1.00) | 0.70(0.58,0.85) |  | 0.88(0.82,0.95) | 0.89(0.75,1.06) | 0.97(0.84,1.12) | 0.56(0.44,0.70) |
| **Coffee** | **Tea** |  |  |  |  |  |  |  |  |  |  |  |  |  |  |
| 0 | 0 | 1.00 (Ref.) | 1.00 (Ref.) | 1.00 (Ref.) | 1.00 (Ref.) |  | 1.00 (Ref.) | 1.00 (Ref.) | 1.00 (Ref.) | 1.00 (Ref.) |  | 1.00 (Ref.) | 1.00 (Ref.) | 1.00 (Ref.) | 1.00 (Ref.) |
| 0 | <1-1 | 1.08(0.90,1.29) | 0.90(0.60,1.34) | 0.91(0.56,1.46) | 1.04(0.62,1.75) |  | 0.85(0.69,1.06) | 0.94(0.57,1.53) | 0.84(0.51,1.38) | 0.96(0.53,1.76) |  | 1.00(0.73,1.38) | 0.81(0.41,1.61) | 0.73(0.35,1.52) | 0.91(0.47,1.77) |
| 0 | 2-4 | 0.94(0.84,1.06) | 0.90(0.69,1.16) | 0.72(0.52,0.99) | 0.84(0.60,1.19) |  | 0.82(0.72,0.94) | 0.91(0.66,1.26) | 0.91(0.66,1.25) | 0.63(0.42,0.95) |  | 0.74(0.60,0.92) | 0.66(0.42,1.04) | 0.68(0.44,1.06) | 0.44(0.27,0.72) |
| 0 | ≥5 | 0.89(0.79,1.00) | 0.77(0.59,1.00) | 0.81(0.59,1.10) | 0.65(0.46,0.93) |  | 0.82(0.72,0.93) | 0.86(0.63,1.19) | 1.00(0.73,1.36) | 0.59(0.40,0.87) |  | 0.77(0.64,0.94) | 0.62(0.41,0.93) | 0.89(0.60,1.32) | 0.30(0.19,0.47) |
| <1-2 | 0 | 1.00(0.87,1.14) | 1.07(0.79,1.44) | 0.69(0.46,1.03) | 0.86(0.57,1.30) |  | 0.89(0.76,1.03) | 0.85(0.59,1.24) | 0.97(0.68,1.39) | 0.74(0.47,1.16) |  | 0.71(0.56,0.91) | 0.51(0.30,0.87) | 0.63(0.38,1.05) | 0.56(0.32,0.95) |
| <1-2 | <1-1 | 0.96(0.84,1.10) | 0.94(0.71,1.26) | 0.84(0.59,1.20) | 0.80(0.54,1.19) |  | 0.84(0.73,0.97) | 0.97(0.68,1.37) | 0.74(0.52,1.05) | 0.71(0.46,1.10) |  | 0.74(0.58,0.93) | 0.57(0.34,0.95) | 0.61(0.37,1.01) | 0.52(0.30,0.87) |
| <1-2 | 2-4 | 0.88(0.78,0.98) | 0.77(0.60,0.98) | 0.74(0.55,1.00) | 0.61(0.44,0.85) |  | 0.75(0.66,0.86) | 0.91(0.67,1.24) | 0.70(0.52,0.95) | 0.52(0.36,0.76) |  | 0.60(0.49,0.73) | 0.45(0.29,0.69) | 0.62(0.41,0.93) | 0.35(0.22,0.54) |
| <1-2 | ≥5 | 0.83(0.74,0.93) | 0.76(0.59,0.98) | 0.67(0.49,0.91) | 0.47(0.33,0.68) |  | 0.73(0.64,0.83) | 0.85(0.62,1.17) | 0.78(0.57,1.06) | 0.44(0.30,0.65) |  | 0.70(0.58,0.85) | 0.60(0.40,0.91) | 0.76(0.51,1.13) | 0.31(0.20,0.48) |
| 3-4 | 0 | 1.00(0.88,1.14) | 1.00(0.75,1.34) | 0.82(0.57,1.18) | 0.57(0.37,0.87) |  | 0.87(0.75,1.00) | 0.92(0.65,1.30) | 1.10(0.79,1.53) | 0.59(0.38,0.91) |  | 0.89(0.72,1.10) | 0.71(0.45,1.13) | 0.96(0.63,1.47) | 0.43(0.26,0.71) |
| 3-4 | <1-1 | 0.89(0.77,1.03) | 0.77(0.56,1.06) | 0.58(0.38,0.89) | 0.63(0.40,1.00) |  | 0.77(0.66,0.90) | 0.89(0.62,1.28) | 0.67(0.46,0.97) | 0.57(0.36,0.91) |  | 0.65(0.52,0.83) | 0.58(0.35,0.96) | 0.70(0.43,1.13) | 0.33(0.18,0.60) |
| 3-4 | 2-4 | 0.87(0.77,0.98) | 0.84(0.65,1.1) | 0.72(0.52,0.99) | 0.53(0.36,0.77) |  | 0.74(0.64,0.84) | 0.83(0.60,1.15) | 0.7(0.51,0.97) | 0.52(0.35,0.78) |  | 0.61(0.50,0.75) | 0.50(0.32,0.78) | 0.69(0.45,1.05) | 0.29(0.18,0.47) |
| 3-4 | ≥5 | 0.90(0.78,1.04) | 0.94(0.68,1.29) | 0.65(0.42,0.99) | 0.65(0.41,1.04) |  | 0.87(0.74,1.01) | 1.00(0.70,1.44) | 1.12(0.79,1.60) | 0.44(0.26,0.74) |  | 0.73(0.59,0.90) | 0.62(0.38,0.99) | 0.76(0.49,1.18) | 0.30(0.17,0.52) |
| ≥5 | 0 | 0.98(0.85,1.12) | 1.00(0.74,1.35) | 0.92(0.64,1.34) | 0.64(0.42,1.00) |  | 0.90(0.78,1.03) | 1.01(0.72,1.42) | 1.11(0.80,1.55) | 0.52(0.33,0.81) |  | 0.84(0.69,1.02) | 0.72(0.47,1.10) | 0.96(0.65,1.44) | 0.39(0.25,0.61) |
| ≥5 | <1-1 | 1.05(0.89,1.23) | 1.09(0.76,1.56) | 0.96(0.61,1.52) | 0.34(0.17,0.70) |  | 0.82(0.70,0.97) | 0.83(0.56,1.24) | 0.97(0.65,1.43) | 0.62(0.37,1.04) |  | 0.65(0.52,0.81) | 0.68(0.42,1.10) | 0.58(0.36,0.94) | 0.28(0.16,0.52) |
| ≥5 | 2-4 | 0.88(0.75,1.03) | 1.15(0.83,1.59) | 0.73(0.46,1.14) | 0.62(0.36,1.04) |  | 0.76(0.65,0.89) | 0.82(0.56,1.20) | 0.95(0.66,1.37) | 0.40(0.23,0.68) |  | 0.73(0.59,0.90) | 0.65(0.41,1.03) | 0.87(0.57,1.33) | 0.32(0.19,0.53) |
| ≥5 | ≥5 | 1.02(0.86,1.21) | 0.98(0.68,1.43) | 1.24(0.81,1.89) | 0.60(0.33,1.09) |  | 0.85(0.72,1.01) | 0.84(0.56,1.27) | 1.03(0.69,1.51) | 0.65(0.38,1.10) |  | 0.83(0.67,1.04) | 0.64(0.39,1.03) | 0.99(0.64,1.53) | 0.40(0.23,0.69) |

Abbreviation: HR, hazard ratio; CI, confidence interval.

Models were adjusted for sex, age, ethnicity, education levels, BMI, alcohol intake frequency, physical activity, dietary pattern, general health status, hypertension, diabetes, and depression. Coffee and tea consumption were mutually adjusted.

**Table S8.** Multivariable HRs and 95% CIs of separate and combined effect of coffee and tea consumption on total and cause-specific mortality by alcohol intake frequency

| Group | | More than one to three times a month | | | |  | Special occasions only or never | | | |
| --- | --- | --- | --- | --- | --- | --- | --- | --- | --- | --- |
|  |  | All causes | Cardiovascular disease | Respiratory disease | Digestive disease |  | All causes | Cardiovascular disease | Respiratory disease | Digestive disease |
| **Coffee** |  |  |  |  |  |  |  |  |  |  |
| 0 |  | 1.00 (Ref.) | 1.00 (Ref.) | 1.00 (Ref.) | 1.00 (Ref.) |  | 1.00 (Ref.) | 1.00 (Ref.) | 1.00 (Ref.) | 1.00 (Ref.) |
| <1-2 |  | 0.88(0.85,0.91) | 0.91(0.85,0.99) | 0.79(0.73,0.86) | 0.75(0.67,0.84) |  | 0.96(0.91,1.01) | 0.98(0.87,1.09) | 0.92(0.82,1.03) | 0.84(0.71,0.99) |
| 3-4 |  | 0.89(0.85,0.92) | 0.92(0.83,1.00) | 0.88(0.79,0.96) | 0.66(0.58,0.76) |  | 1.00(0.93,1.07) | 1.07(0.92,1.23) | 0.94(0.81,1.09) | 0.75(0.60,0.94) |
| ≥5 |  | 0.92(0.88,0.97) | 1.04(0.93,1.16) | 1.01(0.91,1.13) | 0.61(0.52,0.72) |  | 1.05(0.98,1.13) | 1.03(0.87,1.21) | 1.13(0.97,1.32) | 0.79(0.62,1.01) |
| **Tea** |  |  |  |  |  |  |  |  |  |  |
| 0 |  | 1.00 (Ref.) | 1.00 (Ref.) | 1.00 (Ref.) | 1.00 (Ref.) |  | 1.00 (Ref.) | 1.00 (Ref.) | 1.00 (Ref.) | 1.00 (Ref.) |
| <1-1 |  | 0.91(0.87,0.95) | 0.92(0.82,1.03) | 0.77(0.68,0.86) | 0.92(0.79,1.07) |  | 0.94(0.86,1.03) | 0.98(0.82,1.18) | 0.86(0.70,1.06) | 1.00(0.75,1.33) |
| 2-4 |  | 0.82(0.79,0.85) | 0.88(0.80,0.96) | 0.76(0.70,0.84) | 0.66(0.58,0.75) |  | 0.95(0.89,1.01) | 0.86(0.75,0.99) | 0.90(0.77,1.04) | 1.04(0.84,1.28) |
| ≥5 |  | 0.83(0.80,0.86) | 0.86(0.78,0.94) | 0.86(0.78,0.95) | 0.53(0.46,0.61) |  | 0.95(0.89,1.02) | 0.89(0.77,1.03) | 1.01(0.87,1.17) | 0.90(0.72,1.12) |
| **Coffee** | **Tea** |  |  |  |  |  |  |  |  |  |
| 0 | 0 | 1.00 (Ref.) | 1.00 (Ref.) | 1.00 (Ref.) | 1.00 (Ref.) |  | 1.00 (Ref.) | 1.00 (Ref.) | 1.00 (Ref.) | 1.00 (Ref.) |
| 0 | <1-1 | 0.96(0.82,1.13) | 1.02(0.69,1.50) | 0.73(0.49,1.10) | 0.91(0.61,1.37) |  | 1.00(0.82,1.22) | 0.77(0.50,1.18) | 1.06(0.65,1.72) | 1.12(0.60,2.07) |
| 0 | 2-4 | 0.80(0.71,0.89) | 0.93(0.72,1.21) | 0.69(0.54,0.89) | 0.45(0.34,0.60) |  | 0.94(0.83,1.07) | 0.76(0.58,1.00) | 0.92(0.67,1.26) | 1.21(0.81,1.80) |
| 0 | ≥5 | 0.79(0.71,0.87) | 0.83(0.64,1.07) | 0.75(0.59,0.96) | 0.36(0.27,0.47) |  | 0.93(0.83,1.05) | 0.73(0.57,0.95) | 1.18(0.87,1.59) | 0.90(0.61,1.32) |
| <1-2 | 0 | 0.83(0.74,0.93) | 0.94(0.71,1.25) | 0.63(0.47,0.85) | 0.57(0.42,0.78) |  | 1.01(0.86,1.18) | 0.74(0.52,1.05) | 1.11(0.75,1.64) | 1.11(0.68,1.82) |
| <1-2 | <1-1 | 0.80(0.72,0.90) | 0.87(0.66,1.15) | 0.61(0.47,0.81) | 0.54(0.40,0.72) |  | 0.94(0.80,1.11) | 0.99(0.72,1.38) | 0.91(0.60,1.37) | 1.03(0.62,1.71) |
| <1-2 | 2-4 | 0.70(0.63,0.77) | 0.80(0.62,1.02) | 0.55(0.44,0.70) | 0.36(0.28,0.47) |  | 0.91(0.80,1.02) | 0.71(0.55,0.92) | 0.94(0.70,1.28) | 0.90(0.61,1.32) |
| <1-2 | ≥5 | 0.69(0.62,0.76) | 0.79(0.62,1.02) | 0.60(0.47,0.76) | 0.28(0.22,0.37) |  | 0.89(0.78,1.00) | 0.75(0.58,0.97) | 0.98(0.72,1.33) | 0.75(0.51,1.12) |
| 3-4 | 0 | 0.88(0.78,0.98) | 0.95(0.72,1.24) | 0.85(0.65,1.10) | 0.45(0.33,0.60) |  | 0.98(0.84,1.14) | 0.87(0.64,1.19) | 1.13(0.79,1.61) | 0.73(0.44,1.21) |
| 3-4 | <1-1 | 0.73(0.65,0.82) | 0.78(0.59,1.04) | 0.53(0.40,0.71) | 0.42(0.30,0.57) |  | 0.90(0.75,1.08) | 0.91(0.63,1.32) | 0.86(0.54,1.36) | 0.68(0.36,1.28) |
| 3-4 | 2-4 | 0.69(0.62,0.76) | 0.78(0.61,1.01) | 0.57(0.44,0.73) | 0.33(0.25,0.44) |  | 0.93(0.81,1.06) | 0.79(0.59,1.05) | 0.92(0.66,1.3) | 0.80(0.51,1.26) |
| 3-4 | ≥5 | 0.77(0.69,0.87) | 0.92(0.69,1.22) | 0.75(0.57,0.99) | 0.31(0.21,0.44) |  | 0.99(0.85,1.17) | 0.89(0.64,1.25) | 0.99(0.67,1.45) | 0.93(0.56,1.56) |
| ≥5 | 0 | 0.87(0.78,0.97) | 1.01(0.77,1.32) | 0.88(0.68,1.13) | 0.41(0.30,0.55) |  | 1.02(0.89,1.17) | 0.89(0.67,1.19) | 1.22(0.88,1.68) | 0.81(0.52,1.27) |
| ≥5 | <1-1 | 0.79(0.70,0.90) | 1.01(0.75,1.36) | 0.66(0.49,0.90) | 0.33(0.23,0.49) |  | 0.88(0.73,1.07) | 0.67(0.44,1.03) | 1.02(0.66,1.58) | 0.72(0.38,1.37) |
| ≥5 | 2-4 | 0.72(0.64,0.81) | 0.96(0.73,1.28) | 0.70(0.53,0.93) | 0.33(0.23,0.47) |  | 1.02(0.86,1.20) | 0.79(0.55,1.14) | 1.25(0.86,1.82) | 0.71(0.40,1.27) |
| ≥5 | ≥5 | 0.82(0.72,0.94) | 0.86(0.63,1.18) | 0.87(0.65,1.17) | 0.38(0.26,0.56) |  | 1.11(0.93,1.31) | 0.89(0.61,1.28) | 1.3(0.89,1.9) | 1.11(0.65,1.89) |

Abbreviation: HR, hazard ratio; CI, confidence interval.

Models were adjusted for sex, age, ethnicity, education levels, BMI, smoking status, physical activity, dietary pattern, general health status, hypertension, diabetes, and depression. Coffee and tea consumption were mutually adjusted.

**Table S9.** Multivariable HRs and 95% CIs of separate and combined effect of coffee and tea consumption on total and cause-specific mortality by diet pattern

| Group | | Unhealthy diet | | | |  | Healthy diet | | | |
| --- | --- | --- | --- | --- | --- | --- | --- | --- | --- | --- |
|  |  | All causes | Cardiovascular disease | Respiratory disease | Digestive disease |  | All causes | Cardiovascular disease | Respiratory disease | Digestive disease |
| **Coffee** |  |  |  |  |  |  |  |  |  |  |
| 0 |  | 1.00 (Ref.) | 1.00 (Ref.) | 1.00 (Ref.) | 1.00 (Ref.) |  | 1.00 (Ref.) | 1.00 (Ref.) | 1.00 (Ref.) | 1.00 (Ref.) |
| <1-2 |  | 0.90(0.86,0.93) | 0.96(0.88,1.05) | 0.81(0.74,0.88) | 0.76(0.68,0.86) |  | 0.93(0.89,0.97) | 0.91(0.83,1.00) | 0.88(0.79,0.97) | 0.82(0.70,0.95) |
| 3-4 |  | 0.92(0.88,0.96) | 0.98(0.88,1.09) | 0.95(0.86,1.06) | 0.64(0.55,0.74) |  | 0.92(0.88,0.97) | 0.93(0.83,1.05) | 0.83(0.73,0.95) | 0.79(0.66,0.95) |
| ≥5 |  | 0.95(0.91,1.01) | 1.05(0.93,1.18) | 1.06(0.94,1.19) | 0.65(0.55,0.77) |  | 0.98(0.92,1.05) | 1.03(0.89,1.18) | 1.07(0.92,1.23) | 0.71(0.57,0.89) |
| **Tea** |  |  |  |  |  |  |  |  |  |  |
| 0 |  | 1.00 (Ref.) | 1.00 (Ref.) | 1.00 (Ref.) | 1.00 (Ref.) |  | 1.00 (Ref.) | 1.00 (Ref.) | 1.00 (Ref.) | 1.00 (Ref.) |
| <1-1 |  | 0.90(0.85,0.95) | 0.90(0.79,1.01) | 0.73(0.64,0.84) | 1.01(0.85,1.20) |  | 0.96(0.90,1.02) | 1.00(0.86,1.16) | 0.90(0.77,1.07) | 0.86(0.69,1.08) |
| 2-4 |  | 0.85(0.81,0.88) | 0.84(0.76,0.92) | 0.78(0.71,0.87) | 0.78(0.68,0.90) |  | 0.87(0.83,0.92) | 0.94(0.83,1.05) | 0.84(0.74,0.95) | 0.74(0.62,0.88) |
| ≥5 |  | 0.85(0.81,0.89) | 0.83(0.75,0.93) | 0.92(0.83,1.02) | 0.63(0.54,0.74) |  | 0.89(0.84,0.94) | 0.92(0.81,1.04) | 0.89(0.78,1.02) | 0.67(0.56,0.81) |
| **Coffee** | **Tea** |  |  |  |  |  |  |  |  |  |
| 0 | 0 | 1.00 (Ref.) | 1.00 (Ref.) | 1.00 (Ref.) | 1.00 (Ref.) |  | 1.00 (Ref.) | 1.00 (Ref.) | 1.00 (Ref.) | 1.00 (Ref.) |
| 0 | <1-1 | 1.00(0.85,1.19) | 0.85(0.58,1.25) | 0.91(0.60,1.38) | 1.15(0.75,1.75) |  | 0.97(0.80,1.17) | 0.94(0.62,1.44) | 0.83(0.52,1.32) | 0.77(0.43,1.37) |
| 0 | 2-4 | 0.86(0.77,0.96) | 0.78(0.61,1.00) | 0.84(0.64,1.10) | 0.72(0.53,0.97) |  | 0.88(0.78,1.00) | 0.95(0.72,1.26) | 0.75(0.56,1.01) | 0.60(0.42,0.86) |
| 0 | ≥5 | 0.83(0.75,0.92) | 0.74(0.58,0.94) | 0.97(0.75,1.26) | 0.51(0.38,0.69) |  | 0.89(0.79,1.01) | 0.83(0.63,1.09) | 0.85(0.64,1.13) | 0.55(0.39,0.78) |
| <1-2 | 0 | 0.91(0.80,1.03) | 0.88(0.67,1.17) | 0.82(0.60,1.12) | 0.71(0.50,1.00) |  | 0.91(0.79,1.05) | 0.84(0.61,1.17) | 0.77(0.55,1.09) | 0.79(0.53,1.19) |
| <1-2 | <1-1 | 0.84(0.75,0.95) | 0.85(0.65,1.11) | 0.71(0.52,0.96) | 0.73(0.53,1.02) |  | 0.92(0.81,1.06) | 0.92(0.68,1.25) | 0.79(0.57,1.09) | 0.65(0.43,0.97) |
| <1-2 | 2-4 | 0.76(0.68,0.84) | 0.73(0.58,0.92) | 0.69(0.53,0.89) | 0.52(0.39,0.69) |  | 0.82(0.73,0.92) | 0.81(0.62,1.05) | 0.70(0.53,0.92) | 0.50(0.36,0.70) |
| <1-2 | ≥5 | 0.74(0.66,0.82) | 0.73(0.57,0.92) | 0.76(0.59,0.99) | 0.42(0.31,0.56) |  | 0.82(0.73,0.92) | 0.81(0.62,1.06) | 0.72(0.54,0.95) | 0.43(0.31,0.61) |
| 3-4 | 0 | 0.94(0.84,1.06) | 0.92(0.71,1.19) | 1.13(0.85,1.49) | 0.53(0.38,0.75) |  | 0.94(0.82,1.07) | 0.88(0.65,1.19) | 0.80(0.58,1.11) | 0.61(0.41,0.91) |
| 3-4 | <1-1 | 0.75(0.66,0.85) | 0.67(0.51,0.90) | 0.65(0.47,0.90) | 0.56(0.39,0.80) |  | 0.89(0.77,1.03) | 0.95(0.69,1.31) | 0.66(0.46,0.95) | 0.50(0.31,0.79) |
| 3-4 | 2-4 | 0.76(0.68,0.84) | 0.76(0.59,0.97) | 0.72(0.55,0.95) | 0.43(0.31,0.59) |  | 0.79(0.7,0.89) | 0.77(0.58,1.02) | 0.67(0.50,0.91) | 0.53(0.37,0.75) |
| 3-4 | ≥5 | 0.86(0.76,0.98) | 0.83(0.63,1.10) | 1.04(0.78,1.40) | 0.46(0.31,0.67) |  | 0.86(0.75,1.00) | 0.97(0.71,1.34) | 0.65(0.45,0.92) | 0.51(0.33,0.81) |
| ≥5 | 0 | 0.91(0.82,1.02) | 0.91(0.71,1.18) | 1.07(0.82,1.40) | 0.55(0.40,0.76) |  | 0.99(0.87,1.13) | 1.00(0.74,1.35) | 0.98(0.73,1.33) | 0.52(0.35,0.78) |
| ≥5 | <1-1 | 0.84(0.73,0.96) | 0.95(0.71,1.27) | 0.77(0.56,1.08) | 0.44(0.29,0.67) |  | 0.88(0.75,1.04) | 0.80(0.55,1.17) | 0.89(0.61,1.29) | 0.50(0.29,0.85) |
| ≥5 | 2-4 | 0.79(0.70,0.90) | 0.79(0.59,1.05) | 0.95(0.70,1.28) | 0.47(0.32,0.69) |  | 0.86(0.74,1.00) | 1.07(0.77,1.47) | 0.82(0.58,1.16) | 0.44(0.26,0.71) |
| ≥5 | ≥5 | 0.93(0.81,1.06) | 0.85(0.63,1.15) | 1.13(0.83,1.53) | 0.56(0.37,0.84) |  | 0.94(0.80,1.10) | 0.83(0.58,1.20) | 0.96(0.67,1.38) | 0.63(0.38,1.03) |

Abbreviation: HR, hazard ratio; CI, confidence interval.

Models were adjusted for sex, age, ethnicity, education levels, BMI, smoking status, alcohol intake frequency, physical activity, general health status, hypertension, diabetes, and depression. Coffee and tea consumption were mutually adjusted.

**Table S10.** Multivariable HRs and 95% CIs of separate and combined effect of coffee and tea consumption on total and cause-specific mortality by depression

| Group | | No | | | |  | Yes | | | |
| --- | --- | --- | --- | --- | --- | --- | --- | --- | --- | --- |
|  |  | All causes | Cardiovascular disease | Respiratory disease | Digestive disease |  | All causes | Cardiovascular disease | Respiratory disease | Digestive disease |
| **Coffee** |  |  |  |  |  |  |  |  |  |  |
| 0 |  | 1.00 (Ref.) | 1.00 (Ref.) | 1.00 (Ref.) | 1.00 (Ref.) |  | 1.00 (Ref.) | 1.00 (Ref.) | 1.00 (Ref.) | 1.00 (Ref.) |
| <1-2 |  | 0.92(0.89,0.95) | 0.96(0.90,1.03) | 0.83(0.78,0.89) | 0.81(0.73,0.89) |  | 0.84(0.76,0.92) | 0.78(0.63,0.95) | 0.86(0.71,1.04) | 0.63(0.47,0.83) |
| 3-4 |  | 0.93(0.90,0.96) | 0.96(0.89,1.04) | 0.90(0.83,0.99) | 0.73(0.64,0.82) |  | 0.86(0.77,0.97) | 0.98(0.76,1.25) | 0.90(0.70,1.15) | 0.47(0.32,0.69) |
| ≥5 |  | 0.99(0.95,1.03) | 1.06(0.96,1.17) | 1.09(0.99,1.20) | 0.69(0.60,0.80) |  | 0.81(0.71,0.91) | 0.90(0.69,1.18) | 0.88(0.68,1.13) | 0.56(0.39,0.81) |
| **Tea** |  |  |  |  |  |  |  |  |  |  |
| 0 |  | 1.00 (Ref.) | 1.00 (Ref.) | 1.00 (Ref.) | 1.00 (Ref.) |  | 1.00 (Ref.) | 1.00 (Ref.) | 1.00 (Ref.) | 1.00 (Ref.) |
| <1-1 |  | 0.93(0.89,0.97) | 0.93(0.84,1.03) | 0.81(0.73,0.91) | 0.98(0.85,1.14) |  | 0.82(0.71,0.95) | 1.03(0.75,1.41) | 0.68(0.49,0.95) | 0.74(0.48,1.13) |
| 2-4 |  | 0.86(0.83,0.89) | 0.87(0.80,0.94) | 0.83(0.76,0.90) | 0.78(0.70,0.88) |  | 0.80(0.71,0.89) | 0.99(0.78,1.27) | 0.64(0.51,0.81) | 0.63(0.45,0.87) |
| ≥5 |  | 0.87(0.84,0.90) | 0.87(0.80,0.95) | 0.92(0.84,1.00) | 0.67(0.59,0.76) |  | 0.84(0.75,0.94) | 0.84(0.65,1.09) | 0.87(0.69,1.09) | 0.54(0.38,0.75) |
| **Coffee** | **Tea** |  |  |  |  |  |  |  |  |  |
| 0 | 0 | 1.00 (Ref.) | 1.00 (Ref.) | 1.00 (Ref.) | 1.00 (Ref.) |  | 1.00 (Ref.) | 1.00 (Ref.) | 1.00 (Ref.) | 1.00 (Ref.) |
| 0 | <1-1 | 0.99(0.87,1.14) | 0.90(0.66,1.22) | 0.86(0.61,1.20) | 0.99(0.68,1.44) |  | 0.93(0.66,1.32) | 0.86(0.42,1.75) | 0.93(0.41,2.07) | 1.00(0.46,2.21) |
| 0 | 2-4 | 0.87(0.80,0.95) | 0.86(0.71,1.05) | 0.80(0.64,0.99) | 0.68(0.53,0.87) |  | 0.85(0.67,1.08) | 0.82(0.50,1.35) | 0.77(0.45,1.32) | 0.67(0.38,1.18) |
| 0 | ≥5 | 0.86(0.79,0.94) | 0.79(0.65,0.96) | 0.88(0.72,1.08) | 0.53(0.42,0.68) |  | 0.84(0.67,1.05) | 0.76(0.47,1.22) | 1.17(0.71,1.93) | 0.50(0.28,0.87) |
| <1-2 | 0 | 0.91(0.82,1.01) | 0.89(0.71,1.12) | 0.73(0.57,0.94) | 0.77(0.58,1.02) |  | 0.95(0.72,1.26) | 0.69(0.36,1.32) | 1.33(0.73,2.43) | 0.61(0.29,1.25) |
| <1-2 | <1-1 | 0.89(0.81,0.98) | 0.90(0.73,1.12) | 0.73(0.57,0.92) | 0.72(0.54,0.94) |  | 0.77(0.58,1.02) | 0.70(0.38,1.29) | 0.82(0.43,1.56) | 0.64(0.32,1.27) |
| <1-2 | 2-4 | 0.80(0.73,0.86) | 0.77(0.64,0.93) | 0.68(0.55,0.83) | 0.54(0.42,0.68) |  | 0.69(0.55,0.87) | 0.72(0.45,1.16) | 0.74(0.44,1.24) | 0.38(0.21,0.68) |
| <1-2 | ≥5 | 0.79(0.72,0.85) | 0.80(0.66,0.96) | 0.72(0.59,0.89) | 0.44(0.35,0.57) |  | 0.67(0.54,0.85) | 0.52(0.32,0.86) | 0.85(0.51,1.41) | 0.31(0.17,0.57) |
| 3-4 | 0 | 0.95(0.87,1.04) | 0.93(0.75,1.14) | 0.94(0.75,1.18) | 0.57(0.43,0.76) |  | 0.94(0.72,1.22) | 0.78(0.43,1.38) | 1.34(0.76,2.33) | 0.51(0.25,1.02) |
| 3-4 | <1-1 | 0.83(0.75,0.91) | 0.79(0.63,0.99) | 0.65(0.50,0.84) | 0.58(0.43,0.79) |  | 0.58(0.41,0.81) | 0.82(0.42,1.6) | 0.56(0.25,1.24) | 0.17(0.05,0.60) |
| 3-4 | 2-4 | 0.78(0.72,0.85) | 0.77(0.63,0.93) | 0.69(0.56,0.86) | 0.49(0.38,0.63) |  | 0.69(0.54,0.89) | 0.88(0.52,1.48) | 0.71(0.40,1.27) | 0.34(0.17,0.68) |
| 3-4 | ≥5 | 0.88(0.79,0.97) | 0.91(0.72,1.13) | 0.86(0.68,1.09) | 0.52(0.38,0.71) |  | 0.80(0.61,1.07) | 0.82(0.45,1.49) | 0.95(0.52,1.75) | 0.26(0.10,0.66) |
| ≥5 | 0 | 0.97(0.89,1.06) | 0.98(0.80,1.21) | 1.03(0.83,1.28) | 0.54(0.41,0.72) |  | 0.76(0.59,0.98) | 0.79(0.46,1.35) | 1.00(0.58,1.73) | 0.55(0.30,1.02) |
| ≥5 | <1-1 | 0.88(0.79,0.98) | 0.91(0.71,1.16) | 0.80(0.62,1.05) | 0.50(0.36,0.72) |  | 0.68(0.49,0.94) | 0.91(0.47,1.76) | 0.86(0.42,1.73) | 0.18(0.05,0.62) |
| ≥5 | 2-4 | 0.86(0.77,0.95) | 0.93(0.74,1.17) | 0.91(0.72,1.16) | 0.49(0.36,0.68) |  | 0.57(0.42,0.77) | 0.69(0.37,1.28) | 0.71(0.37,1.35) | 0.27(0.11,0.66) |
| ≥5 | ≥5 | 0.93(0.84,1.04) | 0.88(0.68,1.13) | 1.04(0.81,1.34) | 0.58(0.41,0.82) |  | 0.95(0.71,1.25) | 0.70(0.37,1.34) | 1.17(0.65,2.13) | 0.59(0.28,1.22) |

Abbreviation: HR, hazard ratio; CI, confidence interval.

Models were adjusted for sex, age, ethnicity, education levels, BMI, smoking status, alcohol intake frequency, physical activity, dietary pattern, general health status, hypertension, and diabetes. Coffee and tea consumption were mutually adjusted.

**Table S11.** Multivariable HRs and 95% CIs of separate and combined effect of coffee and tea consumption on total and cause-specific mortality by diabetes

| Group | | No | | | |  | Yes | | | |
| --- | --- | --- | --- | --- | --- | --- | --- | --- | --- | --- |
|  |  | All causes | Cardiovascular disease | Respiratory disease | Digestive disease |  | All causes | Cardiovascular disease | Respiratory disease | Digestive disease |
| **Coffee** |  |  |  |  |  |  |  |  |  |  |
| 0 |  | 1.00 (Ref.) | 1.00 (Ref.) | 1.00 (Ref.) | 1.00 (Ref.) |  | 1.00 (Ref.) | 1.00 (Ref.) | 1.00 (Ref.) | 1.00 (Ref.) |
| <1-2 |  | 0.91(0.88,0.93) | 0.94(0.88,1.01) | 0.82(0.76,0.88) | 0.77(0.70,0.86) |  | 0.93(0.86,1.00) | 0.94(0.82,1.07) | 0.97(0.82,1.15) | 0.85(0.69,1.07) |
| 3-4 |  | 0.93(0.90,0.96) | 0.97(0.89,1.06) | 0.91(0.84,1.00) | 0.70(0.62,0.80) |  | 0.86(0.79,0.95) | 0.90(0.76,1.07) | 0.87(0.70,1.08) | 0.67(0.50,0.89) |
| ≥5 |  | 0.96(0.92,1.01) | 1.02(0.92,1.13) | 1.08(0.98,1.19) | 0.65(0.56,0.76) |  | 0.97(0.87,1.07) | 1.11(0.92,1.33) | 0.92(0.72,1.18) | 0.77(0.56,1.07) |
| **Tea** |  |  |  |  |  |  |  |  |  |  |
| 0 |  | 1.00 (Ref.) | 1.00 (Ref.) | 1.00 (Ref.) | 1.00 (Ref.) |  | 1.00 (Ref.) | 1.00 (Ref.) | 1.00 (Ref.) | 1.00 (Ref.) |
| <1-1 |  | 0.93(0.89,0.97) | 0.99(0.89,1.10) | 0.79(0.70,0.88) | 0.97(0.84,1.13) |  | 0.84(0.75,0.95) | 0.76(0.62,0.94) | 0.82(0.62,1.09) | 0.79(0.55,1.14) |
| 2-4 |  | 0.85(0.82,0.88) | 0.88(0.81,0.96) | 0.78(0.72,0.85) | 0.72(0.64,0.82) |  | 0.89(0.82,0.97) | 0.86(0.74,1.00) | 0.91(0.74,1.11) | 0.96(0.74,1.25) |
| ≥5 |  | 0.86(0.83,0.89) | 0.87(0.79,0.95) | 0.89(0.82,0.97) | 0.61(0.53,0.69) |  | 0.91(0.83,1.00) | 0.86(0.73,1.01) | 1.00(0.81,1.24) | 0.86(0.65,1.14) |
| **Coffee** | **Tea** |  |  |  |  |  |  |  |  |  |
| 0 | 0 | 1.00 (Ref.) | 1.00 (Ref.) | 1.00 (Ref.) | 1.00 (Ref.) |  | 1.00 (Ref.) | 1.00 (Ref.) | 1.00 (Ref.) | 1.00 (Ref.) |
| 0 | <1-1 | 1.05(0.92,1.21) | 0.98(0.71,1.36) | 0.93(0.67,1.30) | 1.02(0.71,1.47) |  | 0.72(0.51,1.01) | 0.70(0.40,1.24) | 0.60(0.25,1.44) | 1.01(0.40,2.58) |
| 0 | 2-4 | 0.86(0.79,0.94) | 0.88(0.71,1.09) | 0.76(0.61,0.94) | 0.60(0.47,0.77) |  | 0.92(0.75,1.14) | 0.82(0.57,1.18) | 1.02(0.61,1.71) | 1.18(0.63,2.21) |
| 0 | ≥5 | 0.84(0.77,0.92) | 0.78(0.63,0.97) | 0.87(0.71,1.07) | 0.46(0.36,0.59) |  | 0.97(0.78,1.19) | 0.79(0.55,1.13) | 1.21(0.74,2.00) | 1.07(0.57,1.98) |
| <1-2 | 0 | 0.91(0.82,1.00) | 0.89(0.69,1.14) | 0.74(0.58,0.96) | 0.69(0.52,0.92) |  | 0.95(0.75,1.21) | 0.84(0.55,1.26) | 1.12(0.63,1.98) | 1.13(0.56,2.27) |
| <1-2 | <1-1 | 0.88(0.80,0.97) | 0.95(0.75,1.20) | 0.70(0.55,0.89) | 0.66(0.50,0.87) |  | 0.84(0.66,1.07) | 0.66(0.43,1.01) | 1.00(0.56,1.79) | 0.91(0.44,1.88) |
| <1-2 | 2-4 | 0.77(0.71,0.84) | 0.78(0.63,0.95) | 0.64(0.52,0.79) | 0.45(0.36,0.57) |  | 0.87(0.71,1.07) | 0.75(0.53,1.06) | 1.04(0.64,1.70) | 1.01(0.55,1.85) |
| <1-2 | ≥5 | 0.76(0.70,0.83) | 0.77(0.63,0.95) | 0.70(0.57,0.86) | 0.38(0.30,0.48) |  | 0.85(0.69,1.04) | 0.79(0.55,1.12) | 1.03(0.63,1.70) | 0.82(0.44,1.53) |
| 3-4 | 0 | 0.96(0.87,1.05) | 0.92(0.73,1.16) | 0.98(0.78,1.23) | 0.54(0.41,0.71) |  | 0.90(0.72,1.12) | 0.89(0.61,1.3) | 1.07(0.62,1.84) | 0.76(0.38,1.53) |
| 3-4 | <1-1 | 0.81(0.73,0.89) | 0.83(0.65,1.06) | 0.65(0.50,0.84) | 0.50(0.37,0.68) |  | 0.79(0.61,1.01) | 0.66(0.42,1.04) | 0.60(0.31,1.18) | 0.75(0.34,1.65) |
| 3-4 | 2-4 | 0.77(0.70,0.84) | 0.78(0.63,0.97) | 0.67(0.54,0.83) | 0.42(0.33,0.55) |  | 0.80(0.64,0.99) | 0.73(0.50,1.06) | 0.94(0.56,1.59) | 0.80(0.41,1.55) |
| 3-4 | ≥5 | 0.88(0.80,0.97) | 0.96(0.76,1.22) | 0.85(0.67,1.08) | 0.45(0.33,0.61) |  | 0.76(0.59,0.99) | 0.65(0.41,1.04) | 0.98(0.53,1.81) | 0.60(0.25,1.44) |
| ≥5 | 0 | 0.94(0.86,1.03) | 0.94(0.75,1.18) | 1.03(0.83,1.28) | 0.49(0.37,0.64) |  | 1.01(0.81,1.27) | 1.02(0.70,1.49) | 1.02(0.60,1.75) | 1.01(0.52,1.96) |
| ≥5 | <1-1 | 0.86(0.77,0.96) | 0.93(0.72,1.21) | 0.74(0.57,0.97) | 0.45(0.32,0.64) |  | 0.83(0.62,1.09) | 0.81(0.50,1.31) | 1.28(0.68,2.41) | 0.36(0.12,1.12) |
| ≥5 | 2-4 | 0.83(0.75,0.92) | 0.90(0.70,1.15) | 0.90(0.71,1.15) | 0.42(0.30,0.58) |  | 0.77(0.59,1.00) | 0.91(0.59,1.40) | 0.65(0.33,1.28) | 0.69(0.30,1.60) |
| ≥5 | ≥5 | 0.92(0.82,1.02) | 0.83(0.63,1.09) | 1.03(0.80,1.33) | 0.50(0.35,0.7) |  | 1.06(0.81,1.39) | 0.94(0.59,1.49) | 1.18(0.63,2.20) | 1.26(0.58,2.73) |

Abbreviation: HR, hazard ratio; CI, confidence interval.

Models were adjusted for sex, age, ethnicity, education levels, BMI, smoking status, alcohol intake frequency, physical activity, dietary pattern, general health status, hypertension, and depression. Coffee and tea consumption were mutually adjusted.

**Table S12.** Multivariable HRs and 95% CIs of separate and combined effect of coffee and tea consumption on total and cause-specific mortality by hypertension

| Group | | No | | | |  | Yes | | | |
| --- | --- | --- | --- | --- | --- | --- | --- | --- | --- | --- |
|  |  | All causes | Cardiovascular disease | Respiratory disease | Digestive disease |  | All causes | Cardiovascular disease | Respiratory disease | Digestive disease |
| **Coffee** |  |  |  |  |  |  |  |  |  |  |
| 0 |  | 1.00 (Ref.) | 1.00 (Ref.) | 1.00 (Ref.) | 1.00 (Ref.) |  | 1.00 (Ref.) | 1.00 (Ref.) | 1.00 (Ref.) | 1.00 (Ref.) |
| <1-2 |  | 0.92(0.89,0.96) | 0.96(0.87,1.05) | 0.79(0.72,0.87) | 0.82(0.72,0.94) |  | 0.89(0.86,0.93) | 0.93(0.85,1.01) | 0.89(0.81,0.98) | 0.75(0.66,0.86) |
| 3-4 |  | 0.96(0.92,1) | 0.95(0.85,1.06) | 0.97(0.87,1.08) | 0.77(0.65,0.9) |  | 0.87(0.83,0.92) | 0.97(0.87,1.08) | 0.82(0.73,0.93) | 0.63(0.53,0.74) |
| ≥5 |  | 0.99(0.94,1.05) | 1.04(0.92,1.19) | 1.11(0.98,1.25) | 0.74(0.62,0.89) |  | 0.93(0.88,0.99) | 1.04(0.92,1.17) | 1.00(0.87,1.15) | 0.61(0.49,0.74) |
| **Tea** |  |  |  |  |  |  |  |  |  |  |
| 0 |  | 1.00 (Ref.) | 1.00 (Ref.) | 1.00 (Ref.) | 1.00 (Ref.) |  | 1.00 (Ref.) | 1.00 (Ref.) | 1.00 (Ref.) | 1.00 (Ref.) |
| <1-1 |  | 0.94(0.89,0.99) | 1.02(0.89,1.17) | 0.78(0.68,0.90) | 0.95(0.79,1.15) |  | 0.89(0.84,0.95) | 0.87(0.77,0.99) | 0.81(0.69,0.94) | 0.95(0.78,1.16) |
| 2-4 |  | 0.87(0.83,0.90) | 0.90(0.8,1.01) | 0.85(0.76,0.94) | 0.75(0.65,0.88) |  | 0.84(0.8,0.89) | 0.86(0.78,0.95) | 0.76(0.67,0.85) | 0.77(0.66,0.90) |
| ≥5 |  | 0.88(0.84,0.92) | 0.93(0.82,1.04) | 0.95(0.85,1.06) | 0.66(0.56,0.78) |  | 0.85(0.80,0.90) | 0.82(0.74,0.92) | 0.87(0.77,0.98) | 0.64(0.54,0.76) |
| **Coffee** | **Tea** |  |  |  |  |  |  |  |  |  |
| 0 | 0 | 1.00 (Ref.) | 1.00 (Ref.) | 1.00 (Ref.) | 1.00 (Ref.) |  | 1.00 (Ref.) | 1.00 (Ref.) | 1.00 (Ref.) | 1.00 (Ref.) |
| 0 | <1-1 | 1.05(0.88,1.24) | 0.80(0.51,1.27) | 0.90(0.58,1.38) | 0.99(0.61,1.59) |  | 0.92(0.76,1.11) | 0.95(0.66,1.37) | 0.84(0.53,1.31) | 1.03(0.64,1.67) |
| 0 | 2-4 | 0.90(0.81,1.00) | 0.88(0.67,1.16) | 0.83(0.64,1.09) | 0.60(0.44,0.83) |  | 0.83(0.73,0.94) | 0.84(0.65,1.08) | 0.77(0.57,1.04) | 0.75(0.53,1.05) |
| 0 | ≥5 | 0.88(0.79,0.97) | 0.86(0.66,1.11) | 0.91(0.71,1.17) | 0.50(0.37,0.68) |  | 0.83(0.73,0.94) | 0.72(0.56,0.93) | 0.93(0.70,1.25) | 0.57(0.40,0.79) |
| <1-2 | 0 | 0.91(0.80,1.03) | 0.92(0.67,1.26) | 0.60(0.43,0.84) | 0.71(0.49,1.02) |  | 0.91(0.79,1.05) | 0.83(0.62,1.11) | 1.00(0.72,1.40) | 0.78(0.53,1.15) |
| <1-2 | <1-1 | 0.92(0.81,1.03) | 0.93(0.70,1.25) | 0.69(0.51,0.93) | 0.73(0.51,1.02) |  | 0.83(0.72,0.96) | 0.84(0.64,1.11) | 0.81(0.58,1.13) | 0.68(0.46,0.99) |
| <1-2 | 2-4 | 0.81(0.74,0.90) | 0.81(0.63,1.05) | 0.67(0.52,0.86) | 0.49(0.37,0.66) |  | 0.75(0.66,0.84) | 0.73(0.57,0.93) | 0.72(0.54,0.96) | 0.54(0.39,0.75) |
| <1-2 | ≥5 | 0.82(0.74,0.90) | 0.83(0.64,1.07) | 0.72(0.56,0.93) | 0.42(0.31,0.57) |  | 0.72(0.64,0.81) | 0.72(0.56,0.92) | 0.77(0.58,1.03) | 0.43(0.31,0.60) |
| 3-4 | 0 | 1.03(0.92,1.15) | 0.93(0.70,1.25) | 1.07(0.81,1.41) | 0.60(0.42,0.85) |  | 0.85(0.74,0.97) | 0.89(0.68,1.17) | 0.91(0.66,1.25) | 0.53(0.36,0.79) |
| 3-4 | <1-1 | 0.88(0.78,0.99) | 0.97(0.72,1.31) | 0.67(0.49,0.92) | 0.52(0.36,0.77) |  | 0.71(0.61,0.83) | 0.64(0.47,0.87) | 0.62(0.43,0.90) | 0.56(0.37,0.85) |
| 3-4 | 2-4 | 0.81(0.73,0.90) | 0.74(0.57,0.97) | 0.75(0.58,0.98) | 0.47(0.34,0.65) |  | 0.73(0.64,0.83) | 0.79(0.61,1.02) | 0.64(0.47,0.87) | 0.47(0.33,0.67) |
| 3-4 | ≥5 | 0.91(0.81,1.03) | 0.91(0.67,1.23) | 0.95(0.71,1.26) | 0.51(0.35,0.75) |  | 0.81(0.70,0.94) | 0.89(0.66,1.19) | 0.79(0.55,1.12) | 0.43(0.27,0.68) |
| ≥5 | 0 | 0.98(0.88,1.09) | 0.93(0.70,1.23) | 1.07(0.82,1.39) | 0.54(0.39,0.76) |  | 0.91(0.79,1.04) | 0.98(0.75,1.28) | 0.98(0.72,1.34) | 0.55(0.38,0.80) |
| ≥5 | <1-1 | 0.90(0.79,1.03) | 0.97(0.70,1.35) | 0.77(0.55,1.07) | 0.46(0.29,0.71) |  | 0.80(0.68,0.95) | 0.85(0.61,1.17) | 0.88(0.60,1.28) | 0.46(0.28,0.76) |
| ≥5 | 2-4 | 0.90(0.80,1.02) | 0.99(0.73,1.35) | 0.96(0.72,1.29) | 0.55(0.37,0.80) |  | 0.71(0.61,0.83) | 0.82(0.61,1.11) | 0.79(0.56,1.13) | 0.33(0.20,0.55) |
| ≥5 | ≥5 | 0.97(0.84,1.10) | 0.95(0.68,1.33) | 1.08(0.79,1.46) | 0.55(0.36,0.84) |  | 0.90(0.77,1.06) | 0.78(0.56,1.08) | 1.05(0.74,1.50) | 0.63(0.40,1.00) |

Abbreviation: HR, hazard ratio; CI, confidence interval.

Models were adjusted for sex, age, ethnicity, education levels, BMI, smoking status, alcohol intake frequency, physical activity, dietary pattern, general health status, diabetes, and depression. Coffee and tea consumption were mutually adjusted.

**Table S13.** Multivariable HRs and 95% CIs of separate and combined effect of coffee and tea consumption on total and cause-specific mortality after exclusion of first three years of follow-up

| Group | | All causes | *P* value | Cardiovascular disease | *P* value | Respiratory disease | *P* value | Digestive disease | *P* value |
| --- | --- | --- | --- | --- | --- | --- | --- | --- | --- |
| **Coffee** |  |  |  |  |  |  |  |  |  |
| 0 |  | 1 (Ref.) |  | 1 (Ref.) |  | 1 (Ref.) |  | 1 (Ref.) |  |
| <1-2 |  | 0.91 (0.89,0.94) | <0.001 | 0.95 (0.89,1.02) | 0.153 | 0.83 (0.77,0.89) | <0.001 | 0.82 (0.74,0.90) | <0.001 |
| 3-4 |  | 0.93 (0.90,0.97) | <0.001 | 0.97 (0.89,1.06) | 0.493 | 0.90 (0.82,0.98) | 0.013 | 0.73 (0.64,0.82) | <0.001 |
| ≥5 |  | 0.99 (0.95,1.03) | 0.679 | 1.05 (0.95,1.16) | 0.333 | 1.07 (0.98,1.18) | 0.129 | 0.72 (0.62,0.84) | <0.001 |
| **Tea** |  |  |  |  |  |  |  |  |  |
| 0 |  | 1 (Ref.) |  | 1 (Ref.) |  | 1 (Ref.) |  | 1 (Ref.) |  |
| <1-1 |  | 0.91 (0.87,0.96) | <0.001 | 0.93 (0.84,1.03) | 0.167 | 0.78 (0.70,0.87) | <0.001 | 0.96 (0.83,1.12) | 0.612 |
| 2-4 |  | 0.86 (0.83,0.89) | <0.001 | 0.89 (0.82,0.96) | 0.004 | 0.81 (0.74,0.88) | <0.001 | 0.79 (0.70,0.88) | <0.001 |
| ≥5 |  | 0.88 (0.85,0.91) | <0.001 | 0.87 (0.79,0.94) | 0.001 | 0.91 (0.83,0.99) | 0.028 | 0.69 (0.61,0.79) | <0.001 |
| **Coffee** | **Tea** |  |  |  |  |  |  |  |  |
| 0 | 0 | 1 (Ref.) |  | 1 (Ref.) |  | 1 (Ref.) |  | 1 (Ref.) |  |
| 0 | <1-1 | 0.97 (0.84,1.11) | 0.638 | 0.92 (0.68,1.26) | 0.612 | 0.79 (0.57,1.10) | 0.163 | 1.06 (0.72,1.54) | 0.777 |
| 0 | 2-4 | 0.89 (0.82,0.98) | 0.013 | 0.92 (0.75,1.13) | 0.417 | 0.76 (0.62,0.94) | 0.010 | 0.72 (0.55,0.93) | 0.012 |
| 0 | ≥5 | 0.88 (0.80,0.96) | 0.003 | 0.81 (0.67,0.99) | 0.041 | 0.87 (0.72,1.06) | 0.172 | 0.59 (0.46,0.76) | <0.001 |
| <1-2 | 0 | 0.93 (0.84,1.02) | 0.134 | 0.92 (0.73,1.16) | 0.467 | 0.74 (0.58,0.94) | 0.014 | 0.80 (0.60,1.07) | 0.138 |
| <1-2 | <1-1 | 0.89 (0.81,0.98) | 0.021 | 0.93 (0.74,1.16) | 0.515 | 0.68 (0.54,0.86) | 0.001 | 0.77 (0.58,1.02) | 0.067 |
| <1-2 | 2-4 | 0.80 (0.74,0.87) | <0.001 | 0.82 (0.68,1.00) | 0.045 | 0.66 (0.54,0.80) | <0.001 | 0.58 (0.45,0.74) | <0.001 |
| <1-2 | ≥5 | 0.79 (0.73,0.86) | <0.001 | 0.80 (0.66,0.98) | 0.029 | 0.70 (0.57,0.85) | <0.001 | 0.48 (0.38,0.62) | <0.001 |
| 3-4 | 0 | 0.97 (0.88,1.06) | 0.474 | 0.97 (0.78,1.20) | 0.784 | 0.93 (0.75,1.15) | 0.489 | 0.63 (0.47,0.84) | 0.001 |
| 3-4 | <1-1 | 0.81 (0.73,0.90) | <0.001 | 0.8 (0.63,1.02) | 0.067 | 0.60 (0.47,0.77) | <0.001 | 0.58 (0.43,0.80) | 0.001 |
| 3-4 | 2-4 | 0.79 (0.73,0.87) | <0.001 | 0.82 (0.67,1.00) | 0.053 | 0.66 (0.54,0.81) | <0.001 | 0.51 (0.39,0.67) | <0.001 |
| 3-4 | ≥5 | 0.91 (0.83,1.01) | 0.076 | 0.97 (0.77,1.21) | 0.764 | 0.85 (0.68,1.07) | 0.163 | 0.54 (0.39,0.75) | <0.001 |
| ≥5 | 0 | 0.99 (0.90,1.08) | 0.814 | 1.02 (0.82,1.26) | 0.885 | 1.00 (0.81,1.23) | 0.999 | 0.61 (0.46,0.80) | <0.001 |
| ≥5 | <1-1 | 0.89 (0.80,0.99) | 0.038 | 0.97 (0.76,1.24) | 0.806 | 0.77 (0.60,1.00) | 0.050 | 0.52 (0.36,0.74) | <0.001 |
| ≥5 | 2-4 | 0.85 (0.76,0.94) | 0.002 | 0.91 (0.72,1.15) | 0.441 | 0.86 (0.68,1.08) | 0.198 | 0.50 (0.36,0.70) | <0.001 |
| ≥5 | ≥5 | 0.98 (0.88,1.10) | 0.755 | 0.91 (0.71,1.18) | 0.477 | 1.02 (0.80,1.29) | 0.894 | 0.69 (0.49,0.97) | 0.034 |

Abbreviation: HR, hazard ratio; CI, confidence interval.

Models were adjusted for sex, age, ethnicity, education levels, BMI, smoking status, alcohol intake frequency, physical activity, dietary pattern, general health status, hypertension, diabetes, and depression. Coffee and tea consumption were mutually adjusted.

**Table S14.** Multivariable HRs and 95% CIs of separate and combined effect of coffee and tea consumption on total and cause-specific mortality after exclusion patients with prevalent CVD and cancer at baseline

| Group | | All causes | *P* value | Cardiovascular disease | *P* value | Respiratory disease | *P* value | Digestive disease | *P* value |
| --- | --- | --- | --- | --- | --- | --- | --- | --- | --- |
| **Coffee** |  |  |  |  |  |  |  |  |  |
| 0 |  | 1 (Ref.) |  | 1 (Ref.) |  | 1 (Ref.) |  | 1 (Ref.) |  |
| <1-2 |  | 0.91 (0.88,0.94) | <0.001 | 0.99 (0.91,1.07) | 0.728 | 0.79 (0.73,0.86) | <0.001 | 0.80 (0.71,0.90) | <0.001 |
| 3-4 |  | 0.93 (0.89,0.97) | 0.001 | 1.01 (0.91,1.11) | 0.875 | 0.92 (0.83,1.01) | 0.091 | 0.67 (0.58,0.78) | <0.001 |
| ≥5 |  | 0.99 (0.94,1.04) | 0.644 | 1.08 (0.96,1.21) | 0.197 | 1.09 (0.98,1.22) | 0.122 | 0.67 (0.57,0.79) | <0.001 |
| **Tea** |  |  |  |  |  |  |  |  |  |
| 0 |  | 1 (Ref.) |  | 1 (Ref.) |  | 1 (Ref.) |  | 1 (Ref.) |  |
| <1-1 |  | 0.90 (0.86,0.95) | <0.001 | 0.94 (0.84,1.06) | 0.310 | 0.77 (0.68,0.88) | <0.001 | 0.96 (0.81,1.13) | 0.624 |
| 2-4 |  | 0.85 (0.82,0.89) | <0.001 | 0.86 (0.78,0.95) | 0.002 | 0.81 (0.73,0.89) | <0.001 | 0.78 (0.68,0.89) | <0.001 |
| ≥5 |  | 0.87 (0.83,0.91) | <0.001 | 0.89 (0.81,0.99) | 0.029 | 0.92 (0.83,1.02) | 0.135 | 0.63 (0.54,0.72) | <0.001 |
| **Coffee** | **Tea** |  |  |  |  |  |  |  |  |
| 0 | 0 | 1 (Ref.) |  | 1 (Ref.) |  | 1 (Ref.) |  | 1 (Ref.) |  |
| 0 | <1-1 | 0.91 (0.78,1.07) | 0.251 | 0.87 (0.60,1.25) | 0.444 | 0.68 (0.44,1.04) | 0.078 | 1.06 (0.71,1.61) | 0.767 |
| 0 | 2-4 | 0.86 (0.78,0.95) | 0.004 | 0.82 (0.65,1.04) | 0.096 | 0.81 (0.63,1.04) | 0.105 | 0.72 (0.55,0.96) | 0.024 |
| 0 | ≥5 | 0.85 (0.77,0.93) | 0.001 | 0.81 (0.64,1.02) | 0.073 | 0.97 (0.77,1.24) | 0.823 | 0.50 (0.38,0.66) | <0.001 |
| <1-2 | 0 | 0.90 (0.80,1.01) | 0.064 | 0.93 (0.71,1.21) | 0.577 | 0.71 (0.52,0.95) | 0.023 | 0.77 (0.56,1.07) | 0.119 |
| <1-2 | <1-1 | 0.89 (0.80,0.99) | 0.033 | 0.93 (0.72,1.19) | 0.558 | 0.80 (0.60,1.05) | 0.109 | 0.74 (0.54,1.00) | 0.052 |
| <1-2 | 2-4 | 0.77 (0.70,0.84) | <0.001 | 0.77 (0.62,0.96) | 0.021 | 0.67 (0.53,0.86) | 0.001 | 0.54 (0.41,0.70) | <0.001 |
| <1-2 | ≥5 | 0.77 (0.70,0.84) | <0.001 | 0.83 (0.66,1.04) | 0.098 | 0.71 (0.56,0.90) | 0.005 | 0.44 (0.33,0.58) | <0.001 |
| 3-4 | 0 | 0.95 (0.85,1.06) | 0.343 | 0.97 (0.76,1.24) | 0.808 | 1.04 (0.80,1.35) | 0.78 | 0.54 (0.39,0.75) | <0.001 |
| 3-4 | <1-1 | 0.78 (0.69,0.87) | <0.001 | 0.82 (0.63,1.07) | 0.143 | 0.62 (0.46,0.84) | 0.002 | 0.56 (0.40,0.78) | 0.001 |
| 3-4 | 2-4 | 0.77 (0.70,0.85) | <0.001 | 0.80 (0.63,1.01) | 0.061 | 0.73 (0.57,0.94) | 0.013 | 0.45 (0.33,0.60) | <0.001 |
| 3-4 | ≥5 | 0.87 (0.77,0.97) | 0.015 | 0.89 (0.68,1.16) | 0.385 | 0.93 (0.71,1.23) | 0.621 | 0.53 (0.37,0.75) | <0.001 |
| ≥5 | 0 | 0.95 (0.86,1.05) | 0.336 | 0.95 (0.74,1.21) | 0.666 | 1.11 (0.86,1.43) | 0.421 | 0.57 (0.42,0.78) | <0.001 |
| ≥5 | <1-1 | 0.83 (0.73,0.94) | 0.003 | 0.93 (0.70,1.24) | 0.625 | 0.78 (0.57,1.06) | 0.114 | 0.42 (0.28,0.64) | <0.001 |
| ≥5 | 2-4 | 0.87 (0.77,0.98) | 0.017 | 0.98 (0.75,1.28) | 0.878 | 0.92 (0.69,1.21) | 0.546 | 0.53 (0.38,0.76) | <0.001 |
| ≥5 | ≥5 | 0.93 (0.82,1.06) | 0.288 | 0.88 (0.65,1.19) | 0.408 | 1.15 (0.86,1.53) | 0.358 | 0.48 (0.32,0.73) | 0.001 |

Abbreviation: HR, hazard ratio; CI, confidence interval.

Models were adjusted for sex, age, ethnicity, education levels, BMI, smoking status, alcohol intake frequency, physical activity, dietary pattern, general health status, hypertension, diabetes, and depression. Coffee and tea consumption were mutually adjusted.

**Table S15.** Multivariable HRs and 95% CIs of separate and combined effect of coffee and tea consumption on total and cause-specific mortality adjusted for pack-years categories of cigarette smoking at baseline

| Group | | All causes | *P* value | Cardiovascular disease | *P* value | Respiratory disease | *P* value | Digestive disease | *P* value |
| --- | --- | --- | --- | --- | --- | --- | --- | --- | --- |
| **Coffee** |  |  |  |  |  |  |  |  |  |
| 0 |  | 1 (Ref.) |  | 1 (Ref.) |  | 1 (Ref.) |  | 1 (Ref.) |  |
| <1-2 |  | 0.92 (0.90,0.95) | <0.001 | 0.95 (0.89,1.01) | 0.102 | 0.85 (0.80,0.91) | <0.001 | 0.79 (0.72,0.87) | <0.001 |
| 3-4 |  | 0.95 (0.92,0.98) | 0.002 | 0.98 (0.91,1.06) | 0.653 | 0.95 (0.88,1.03) | 0.248 | 0.72 (0.64,0.81) | <0.001 |
| ≥5 |  | 1.02 (0.98,1.06) | 0.374 | 1.10 (1.00,1.20) | 0.042 | 1.15 (1.05,1.26) | 0.002 | 0.73 (0.63,0.83) | <0.001 |
| **Tea** |  |  |  |  |  |  |  |  |  |
| 0 |  | 1 (Ref.) |  | 1 (Ref.) |  | 1 (Ref.) |  | 1 (Ref.) |  |
| <1-1 |  | 0.93 (0.89,0.97) | <0.001 | 0.94 (0.85,1.03) | 0.191 | 0.80 (0.72,0.89) | <0.001 | 0.96 (0.83,1.10) | 0.522 |
| 2-4 |  | 0.86 (0.83,0.89) | <0.001 | 0.88 (0.81,0.95) | 0.001 | 0.81 (0.75,0.88) | <0.001 | 0.77 (0.69,0.85) | <0.001 |
| ≥5 |  | 0.88 (0.85,0.92) | <0.001 | 0.88 (0.81,0.95) | 0.002 | 0.94 (0.87,1.02) | 0.128 | 0.67 (0.59,0.75) | <0.001 |
| **Coffee** | **Tea** |  |  |  |  |  |  |  |  |
| 0 | 0 | 1 (Ref.) |  | 1 (Ref.) |  | 1 (Ref.) |  | 1 (Ref.) |  |
| 0 | <1-1 | 0.99 (0.87,1.12) | 0.860 | 0.89 (0.67,1.19) | 0.441 | 0.86 (0.63,1.17) | 0.331 | 1.00 (0.71,1.40) | 0.997 |
| 0 | 2-4 | 0.87 (0.80,0.94) | 0.001 | 0.86 (0.71,1.03) | 0.101 | 0.80 (0.66,0.98) | 0.028 | 0.67 (0.53,0.84) | 0.001 |
| 0 | ≥5 | 0.87 (0.81,0.95) | 0.001 | 0.80 (0.67,0.96) | 0.015 | 0.95 (0.79,1.15) | 0.607 | 0.54 (0.43,0.68) | <0.001 |
| <1-2 | 0 | 0.92 (0.84,1.01) | 0.082 | 0.88 (0.71,1.09) | 0.247 | 0.81 (0.64,1.02) | 0.071 | 0.75 (0.57,0.98) | 0.032 |
| <1-2 | <1-1 | 0.90 (0.82,0.98) | 0.017 | 0.89 (0.73,1.10) | 0.281 | 0.77 (0.61,0.96) | 0.021 | 0.71 (0.55,0.92) | 0.009 |
| <1-2 | 2-4 | 0.80 (0.74,0.86) | <0.001 | 0.78 (0.65,0.92) | 0.004 | 0.72 (0.59,0.86) | <0.001 | 0.52 (0.42,0.65) | <0.001 |
| <1-2 | ≥5 | 0.79 (0.73,0.86) | <0.001 | 0.79 (0.66,0.94) | 0.008 | 0.77 (0.64,0.94) | 0.008 | 0.44 (0.35,0.55) | <0.001 |
| 3-4 | 0 | 0.96 (0.88,1.05) | 0.410 | 0.93 (0.76,1.13) | 0.477 | 1.03 (0.84,1.27) | 0.761 | 0.58 (0.45,0.75) | <0.001 |
| 3-4 | <1-1 | 0.83 (0.76,0.91) | <0.001 | 0.81 (0.65,1.00) | 0.051 | 0.68 (0.54,0.87) | 0.002 | 0.56 (0.42,0.74) | <0.001 |
| 3-4 | 2-4 | 0.80 (0.74,0.87) | <0.001 | 0.79 (0.66,0.95) | 0.012 | 0.75 (0.61,0.91) | 0.004 | 0.48 (0.38,0.62) | <0.001 |
| 3-4 | ≥5 | 0.91 (0.83,1.00) | 0.050 | 0.94 (0.76,1.16) | 0.538 | 0.96 (0.77,1.20) | 0.723 | 0.51 (0.38,0.68) | <0.001 |
| ≥5 | 0 | 1.00 (0.92,1.09) | 0.948 | 1.01 (0.84,1.23) | 0.899 | 1.12 (0.92,1.37) | 0.254 | 0.59 (0.46,0.76) | <0.001 |
| ≥5 | <1-1 | 0.90 (0.82,1.00) | 0.051 | 0.95 (0.76,1.19) | 0.657 | 0.88 (0.68,1.12) | 0.294 | 0.49 (0.35,0.68) | <0.001 |
| ≥5 | 2-4 | 0.87 (0.79,0.96) | 0.004 | 0.95 (0.77,1.18) | 0.657 | 0.97 (0.78,1.22) | 0.810 | 0.49 (0.36,0.67) | <0.001 |
| ≥5 | ≥5 | 1.00 (0.90,1.11) | 0.995 | 0.91 (0.72,1.15) | 0.446 | 1.18 (0.93,1.48) | 0.168 | 0.64 (0.47,0.88) | 0.005 |

Abbreviation: HR, hazard ratio; CI, confidence interval.

Models were adjusted for sex, age, ethnicity, education levels, BMI, pack-years categories of cigarette smoking (nonsmokers: having smoked zero pack-years; light smokers: fewer than 20 pack-years; and heavy smokers: 20 or more pack-years), alcohol intake frequency, physical activity, dietary pattern, general health status, hypertension, diabetes, and depression. Coffee and tea consumption were mutually adjusted.

**Table S16.** Multivariable HRs and 95% CIs of separate and combined effect of coffee and tea consumption on total and cause-specific mortality unadjusted for depression at baseline

| Group | | All causes | *P* value | Cardiovascular disease | *P* value | Respiratory disease | *P* value | Digestive disease | *P* value |
| --- | --- | --- | --- | --- | --- | --- | --- | --- | --- |
| **Coffee** |  |  |  |  |  |  |  |  |  |
| 0 |  | 1 (Ref.) |  | 1 (Ref.) |  | 1 (Ref.) |  | 1 (Ref.) |  |
| <1-2 |  | 0.91 (0.88,0.93) | <0.001 | 0.94 (0.88,1.00) | 0.057 | 0.84 (0.78,0.89) | <0.001 | 0.78 (0.71,0.86) | <0.001 |
| 3-4 |  | 0.92 (0.89,0.95) | <0.001 | 0.96 (0.89,1.04) | 0.283 | 0.90 (0.83,0.98) | 0.014 | 0.69 (0.62,0.78) | <0.001 |
| ≥5 |  | 0.97 (0.93,1.01) | 0.095 | 1.04 (0.95,1.14) | 0.377 | 1.06 (0.97,1.16) | 0.196 | 0.67 (0.59,0.77) | <0.001 |
| **Tea** |  |  |  |  |  |  |  |  |  |
| 0 |  | 1 (Ref.) |  | 1 (Ref.) |  | 1 (Ref.) |  | 1 (Ref.) |  |
| <1-1 |  | 0.92 (0.88,0.96) | <0.001 | 0.94 (0.85,1.03) | 0.187 | 0.80 (0.72,0.88) | <0.001 | 0.95 (0.83,1.09) | 0.494 |
| 2-4 |  | 0.86 (0.83,0.88) | <0.001 | 0.88 (0.81,0.94) | 0.001 | 0.80 (0.74,0.87) | <0.001 | 0.76 (0.68,0.85) | <0.001 |
| ≥5 |  | 0.87 (0.84,0.90) | <0.001 | 0.87 (0.80,0.94) | <0.001 | 0.91 (0.84,0.99) | 0.025 | 0.65 (0.58,0.73) | <0.001 |
| **Coffee** | **Tea** |  |  |  |  |  |  |  |  |
| 0 | 0 | 1 (Ref.) |  | 1 (Ref.) |  | 1 (Ref.) |  | 1 (Ref.) |  |
| 0 | <1-1 | 0.99 (0.87,1.12) | 0.882 | 0.90 (0.68,1.19) | 0.454 | 0.86 (0.63,1.18) | 0.353 | 1 (0.71,1.4) | 0.993 |
| 0 | 2-4 | 0.87 (0.80,0.94) | 0.001 | 0.86 (0.71,1.03) | 0.100 | 0.80 (0.65,0.97) | 0.025 | 0.67 (0.53,0.84) | 0.001 |
| 0 | ≥5 | 0.86 (0.79,0.93) | <0.001 | 0.78 (0.65,0.94) | 0.007 | 0.92 (0.76,1.11) | 0.374 | 0.52 (0.42,0.66) | <0.001 |
| <1-2 | 0 | 0.91 (0.83,1.00) | 0.050 | 0.87 (0.70,1.08) | 0.199 | 0.79 (0.63,1.00) | 0.051 | 0.74 (0.57,0.96) | 0.024 |
| <1-2 | <1-1 | 0.88 (0.80,0.96) | 0.004 | 0.88 (0.72,1.08) | 0.218 | 0.74 (0.59,0.92) | 0.008 | 0.70 (0.54,0.90) | 0.005 |
| <1-2 | 2-4 | 0.78 (0.73,0.85) | <0.001 | 0.76 (0.64,0.91) | 0.003 | 0.69 (0.57,0.83) | <0.001 | 0.51 (0.41,0.63) | <0.001 |
| <1-2 | ≥5 | 0.77 (0.71,0.84) | <0.001 | 0.77 (0.64,0.91) | 0.003 | 0.74 (0.61,0.89) | 0.002 | 0.42 (0.34,0.53) | <0.001 |
| 3-4 | 0 | 0.94 (0.87,1.03) | 0.202 | 0.91 (0.75,1.11) | 0.341 | 0.99 (0.80,1.21) | 0.895 | 0.56 (0.43,0.72) | <0.001 |
| 3-4 | <1-1 | 0.80 (0.73,0.88) | <0.001 | 0.78 (0.63,0.97) | 0.025 | 0.65 (0.51,0.82) | <0.001 | 0.53 (0.40,0.71) | <0.001 |
| 3-4 | 2-4 | 0.77 (0.71,0.84) | <0.001 | 0.77 (0.64,0.92) | 0.005 | 0.70 (0.57,0.85) | <0.001 | 0.46 (0.37,0.59) | <0.001 |
| 3-4 | ≥5 | 0.87 (0.79,0.95) | 0.003 | 0.89 (0.72,1.10) | 0.294 | 0.87 (0.70,1.09) | 0.239 | 0.47 (0.35,0.64) | <0.001 |
| ≥5 | 0 | 0.95 (0.87,1.03) | 0.215 | 0.96 (0.79,1.16) | 0.641 | 1.03 (0.84,1.26) | 0.796 | 0.54 (0.42,0.69) | <0.001 |
| ≥5 | <1-1 | 0.86 (0.78,0.95) | 0.004 | 0.90 (0.72,1.14) | 0.384 | 0.81 (0.63,1.03) | 0.091 | 0.46 (0.33,0.63) | <0.001 |
| ≥5 | 2-4 | 0.82 (0.75,0.91) | <0.001 | 0.90 (0.73,1.11) | 0.329 | 0.89 (0.71,1.12) | 0.315 | 0.45 (0.34,0.61) | <0.001 |
| ≥5 | ≥5 | 0.94 (0.85,1.04) | 0.220 | 0.85 (0.68,1.08) | 0.187 | 1.06 (0.84,1.34) | 0.628 | 0.58 (0.43,0.80) | 0.001 |

Abbreviation: HR, hazard ratio; CI, confidence interval.

Models were adjusted for sex, age, ethnicity, education levels, BMI, smoking status, alcohol intake frequency, physical activity, dietary pattern, general health status, hypertension, and diabetes. Coffee and tea consumption were mutually adjusted.

**Table S17.** Multivariable HRs and 95% CIs of separate and combined effect of coffee and tea consumption on total and cause-specific mortality using unimputed data

| Group |  | All causes | *P* value | Cardiovascular disease | *P* value | Respiratory disease | *P* value | Digestive disease | *P* value |
| --- | --- | --- | --- | --- | --- | --- | --- | --- | --- |
| **Coffee** |  |  |  |  |  |  |  |  |  |
| 0 |  | 1 (Ref.) |  | 1 (Ref.) |  | 1 (Ref.) |  | 1 (Ref.) |  |
| <1-2 |  | 0.92 (0.89,0.95) | <0.001 | 0.97 (0.89,1.05) | 0.440 | 0.81 (0.75,0.88) | <0.001 | 0.75 (0.67,0.84) | <0.001 |
| 3-4 |  | 0.92 (0.88,0.96) | <0.001 | 0.97 (0.88,1.07) | 0.500 | 0.88 (0.79,0.97) | 0.014 | 0.67 (0.58,0.78) | <0.001 |
| ≥5 |  | 0.98 (0.93,1.03) | 0.432 | 1.07 (0.95,1.20) | 0.252 | 1.08 (0.97,1.21) | 0.162 | 0.63 (0.53,0.75) | <0.001 |
| **Tea** |  |  |  |  |  |  |  |  |  |
| 0 |  | 1 (Ref.) |  | 1 (Ref.) |  | 1 (Ref.) |  | 1 (Ref.) |  |
| <1-1 |  | 0.94 (0.89,0.99) | 0.016 | 1.03 (0.92,1.16) | 0.562 | 0.79 (0.69,0.90) | <0.001 | 0.94 (0.80,1.12) | 0.502 |
| 2-4 |  | 0.85 (0.82,0.89) | <0.001 | 0.89 (0.81,0.98) | 0.021 | 0.83 (0.75,0.92) | <0.001 | 0.76 (0.66,0.87) | <0.001 |
| ≥5 |  | 0.88 (0.84,0.92) | <0.001 | 0.93 (0.84,1.02) | 0.132 | 0.93 (0.84,1.04) | 0.193 | 0.65 (0.56,0.75) | <0.001 |
| **Coffee** | **Tea** |  |  |  |  |  |  |  |  |
| 0 | 0 | 1 (Ref.) |  | 1 (Ref.) |  | 1 (Ref.) |  | 1 (Ref.) |  |
| 0 | <1-1 | 1.07 (0.91,1.25) | 0.417 | 1.14 (0.80,1.63) | 0.474 | 0.63 (0.41,0.97) | 0.037 | 1.13 (0.73,1.74) | 0.588 |
| 0 | 2-4 | 0.90 (0.81,1.00) | 0.040 | 0.93 (0.73,1.19) | 0.575 | 0.76 (0.60,0.98) | 0.033 | 0.79 (0.59,1.07) | 0.124 |
| 0 | ≥5 | 0.90 (0.82,1.00) | 0.048 | 0.92 (0.73,1.17) | 0.506 | 0.88 (0.69,1.11) | 0.283 | 0.64 (0.48,0.85) | 0.003 |
| <1-2 | 0 | 0.95 (0.84,1.07) | 0.374 | 1.00 (0.76,1.32) | 0.996 | 0.68 (0.51,0.91) | 0.010 | 0.91 (0.66,1.27) | 0.588 |
| <1-2 | <1-1 | 0.92 (0.83,1.03) | 0.148 | 1.04 (0.80,1.35) | 0.767 | 0.66 (0.50,0.88) | 0.004 | 0.78 (0.56,1.08) | 0.135 |
| <1-2 | 2-4 | 0.82 (0.75,0.91) | <0.001 | 0.88 (0.69,1.10) | 0.261 | 0.64 (0.51,0.81) | <0.001 | 0.56 (0.43,0.75) | <0.001 |
| <1-2 | ≥5 | 0.83 (0.75,0.91) | <0.001 | 0.91 (0.72,1.15) | 0.443 | 0.69 (0.54,0.87) | 0.002 | 0.46 (0.34,0.62) | <0.001 |
| 3-4 | 0 | 0.99 (0.89,1.11) | 0.892 | 1.02 (0.79,1.32) | 0.890 | 0.86 (0.66,1.12) | 0.260 | 0.63 (0.45,0.88) | 0.006 |
| 3-4 | <1-1 | 0.87 (0.77,0.97) | 0.014 | 0.95 (0.72,1.25) | 0.718 | 0.61 (0.45,0.82) | 0.001 | 0.63 (0.44,0.89) | 0.010 |
| 3-4 | 2-4 | 0.80 (0.72,0.88) | <0.001 | 0.86 (0.67,1.09) | 0.210 | 0.66 (0.51,0.84) | 0.001 | 0.51 (0.38,0.70) | <0.001 |
| 3-4 | ≥5 | 0.90 (0.80,1.01) | 0.068 | 1.01 (0.77,1.33) | 0.941 | 0.83 (0.63,1.09) | 0.177 | 0.57 (0.39,0.82) | 0.003 |
| ≥5 | 0 | 1.01 (0.91,1.12) | 0.863 | 1.05 (0.82,1.36) | 0.694 | 1.01 (0.79,1.29) | 0.959 | 0.60 (0.44,0.83) | 0.002 |
| ≥5 | <1-1 | 0.94 (0.83,1.07) | 0.333 | 1.16 (0.87,1.54) | 0.327 | 0.78 (0.57,1.06) | 0.106 | 0.45 (0.30,0.70) | <0.001 |
| ≥5 | 2-4 | 0.84 (0.75,0.95) | 0.006 | 1.04 (0.79,1.37) | 0.777 | 0.84 (0.63,1.10) | 0.208 | 0.55 (0.38,0.81) | 0.002 |
| ≥5 | ≥5 | 0.98 (0.86,1.12) | 0.782 | 0.95 (0.70,1.30) | 0.765 | 1.00 (0.74,1.34) | 0.988 | 0.60 (0.39,0.91) | 0.017 |

Abbreviation: HR, hazard ratio; CI, confidence interval.

Models were adjusted for sex, age, ethnicity, education levels, BMI, smoking status, alcohol intake frequency, physical activity, dietary pattern, general health status, hypertension, diabetes, and depression. Coffee and tea consumption were mutually adjusted.


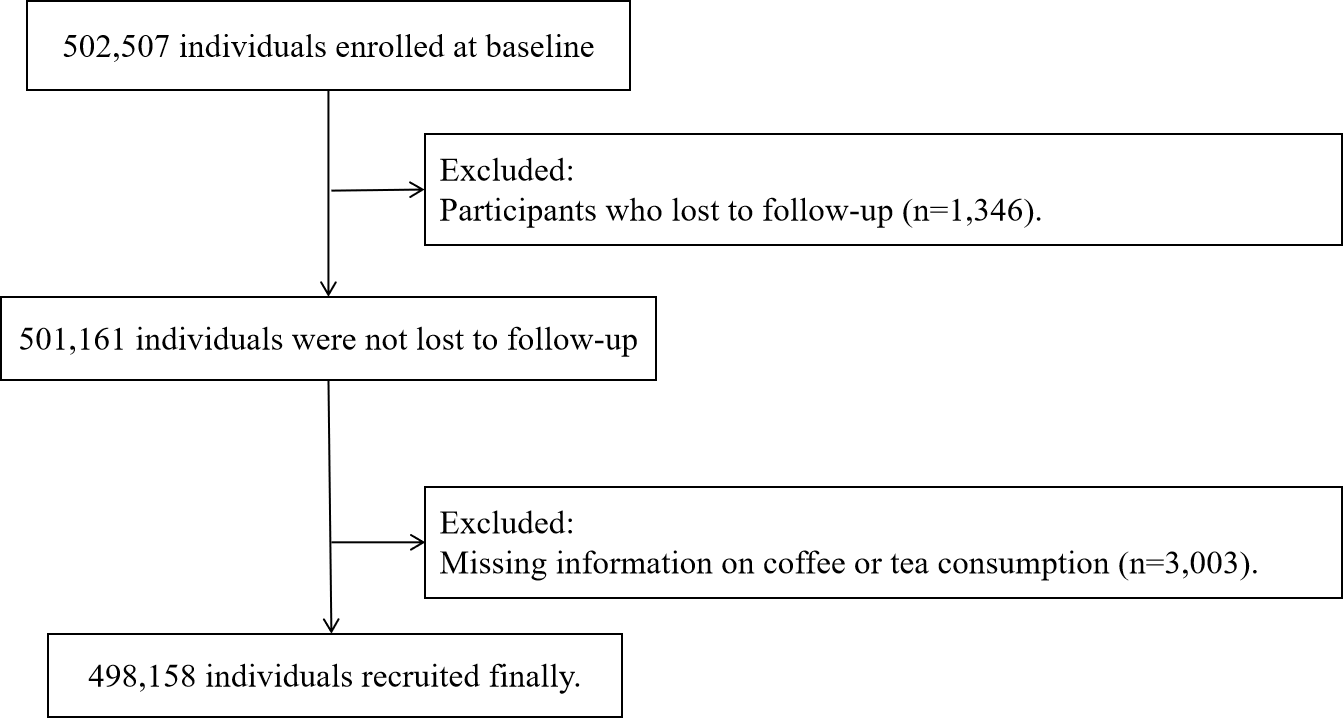


**Fig. S1.** Flowchart for the selection of the analyzed study sample from the UK Biobank Study.


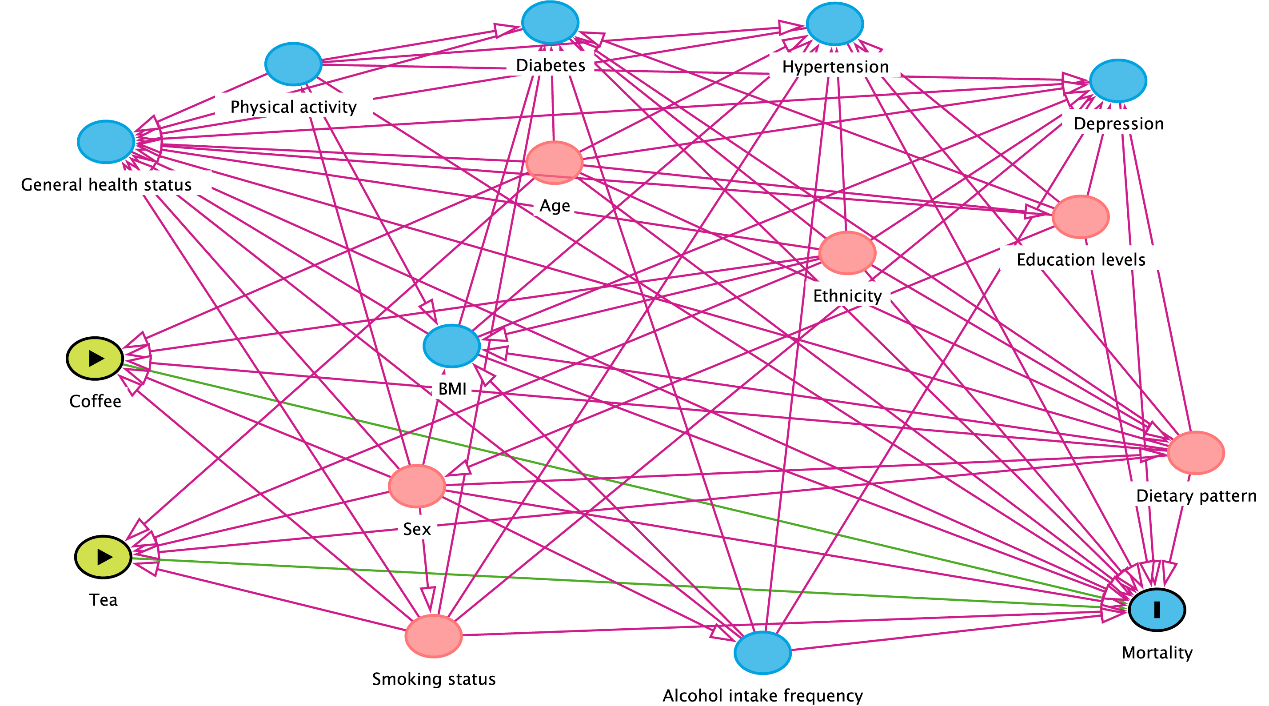


**Fig. S2.** Directed acyclic graph (DAG) derived from previous literature and expert knowledge. Arrows represent causal associations

Coffee and tea are exposure, and mortality is the outcome. BMI, body mass index.


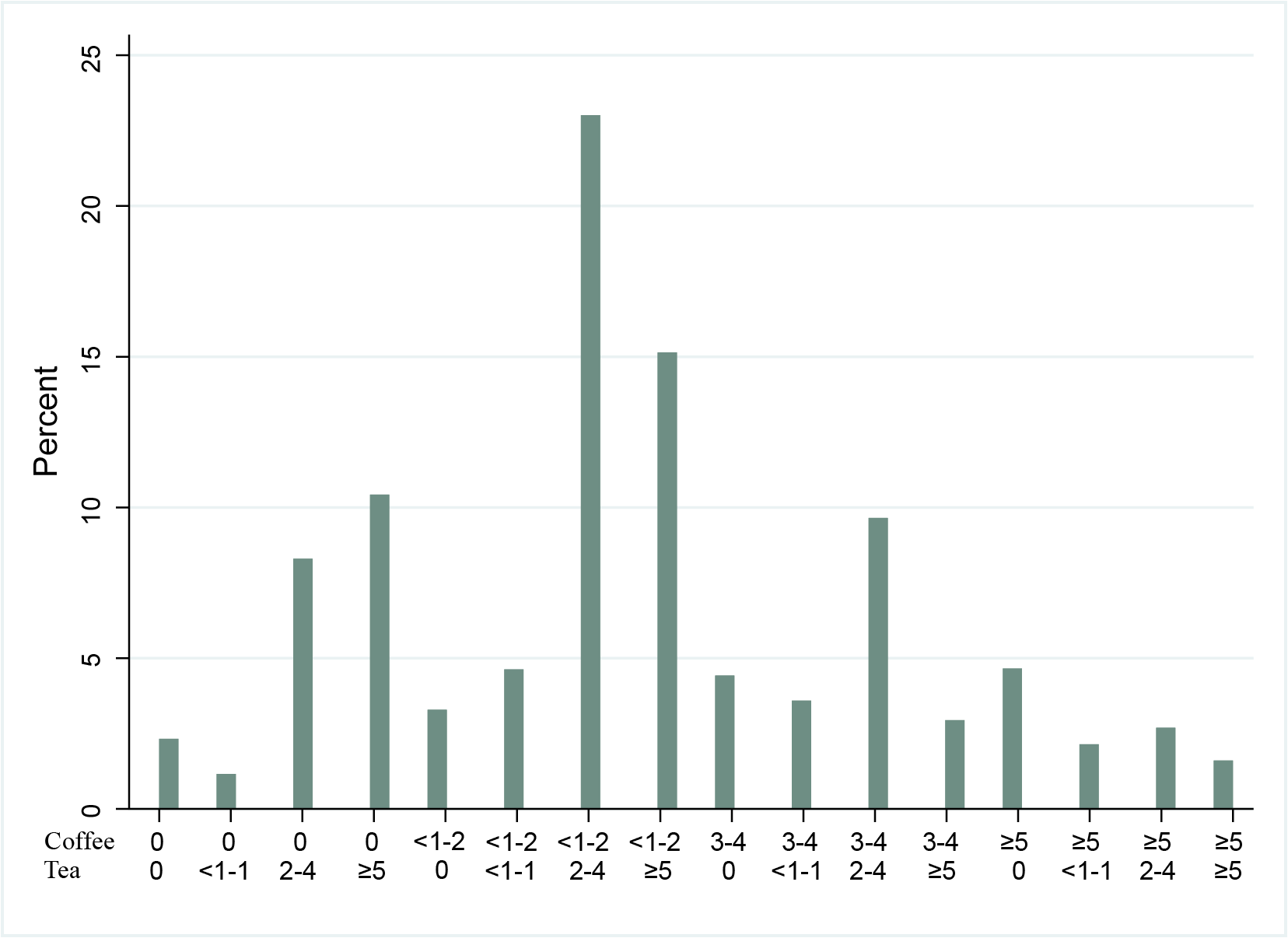


**Fig. S3.** The distribution of combination of coffee and tea consumption
